# Defect‐Rich Porous Cu with Abundant Cu(100) for Acidic CO_2_ Electroreduction in Membrane Electrode Assembly

**DOI:** 10.1002/advs.202510161

**Published:** 2025-07-26

**Authors:** Qiang Fang, Yunzhen Jia, Xuelei Lang, Geng Li, Tao Zhao, Dazhong Zhong, Jinping Li, Qiang Zhao

**Affiliations:** ^1^ College of Chemistry and Chemical Engineering Shanxi Key Laboratory of Gas Energy Efficient and Clean Utilization Taiyuan University of Technology Taiyuan Shanxi 030024 P. R. China

**Keywords:** acidic electrolyte, carbon dioxide reduction, C‐C coupling, membrane electrode assembly, reaction microenvironment

## Abstract

Acidic electrocatalytic CO_2_ reduction (CO_2_RR) faces slow C‐C coupling kinetics and dominant hydrogen evolution, resulting in low C_2+_ yields and selectivity. Here, the porous copper nanosheets (pCu NS) are reported with abundant Cu(100) and defect sites for efficient acidic CO_2_RR to C_2+_ products. In a membrane electrode assembly (MEA), the pCu NS electrodes achieved a remarkable 75.01% C_2+_ production Faradaic efficiency (FE) at a current density of 300 mA cm^−2^ with a full‐cell voltage of 3.8 V. A CO_2_ single‐pass conversion efficiency of up to 74.38% is achieved. In situ Raman spectra and density functional theory calculations revealed that pCu NS not only gives abundant nanopores and defect sites but also preferentially exposes Cu(100) facets, synergistically creating local alkaline microenvironment, maximizing the *CO intermediate coverage, and promoting *CO hydrogenation for C_2+_ production. This work offers a crucial insight for designing an efficient catalyst for efficient acidic CO_2_‐to‐C_2+_ conversion.

## Graphical abstract

This study introduces defect‐rich porous Cu nanosheets (pCu NS) with abundant Cu(100) facets for efficient acidic CO_2_RR to C_2+_ products. The porous structure creates a localized alkaline microenvironment that hinders HER, defects enhance *CO coverage, and the Cu(100) facet facilitates *CO hydrogenation to *COH, demonstrating the essential role of facet/defect engineering in optimizing reaction pathways for C_2+_ products.

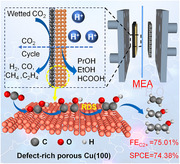

## 1 Introduction

Electrocatalytic CO_2_ reduction reaction (CO_2_RR) has emerged as a promising solution for converting greenhouse gas emissions into valuable chemicals and fuels.^[^ ^1^, ^2^, ^3^ ^]^ However, the efficient and selective generation of multi‐carbon products with high economic value, such as ethylene(C_2_H_4_), ethanol (C_2_H_6_O), and n‐propanol, via CO_2_ reduction remains a significant challenge.^[^ ^4^, ^5^ ^]^ An alkaline/neutral media CO_2_RR, as it curbs the hydrogen evolution reaction (HER) and stimulates C‐C coupling reactions on Cu‐based catalysts, which is considered a pivotal step in the production of multi‐carbon products.^[^ ^6^, ^7^, ^8^ ^]^ Nevertheless, CO_2_ can readily react with local/bulk OH⁻ species to generate (bi)carbonate (CO_3_^2−^ or HCO_3_^−^), which traverses the anion exchange membrane to the anode and is subsequently transformed back to CO₂ in the anode tail gas, resulting in a loss of CO_2_ and a relatively low CO_2_ single pass conversion efficiency (SPCE, <25%).^[^ ^9^, ^10^ ^]^ Furthermore, carbonate formation can also block the gas diffusion electrode and increase the cell resistance. Alternatively, electrocatalytic CO_2_‐to‐C_2+_ products in an acidic system can solve the above problems well.^[^ ^11^, ^12^ ^]^ However, the high concentration of H^+^ in acidic electrolytes is beneficial for the competition HER and hinders the CO_2_RR process.

Copper‐based catalysts are unique catalysts that enable C–C coupling during CO_2_RR, yet their performance is highly dependent on crystallographic orientation and surface atomic arrangements.^[^ ^13^ ^]^ Among Cu facets, the Cu(100) surface has gained significant attention due to its intrinsic ability for the production of C_2+_ products compared to Cu(111).^[^ ^14^ ^]^ Nonetheless, the *CO coverage on well‐defined Cu(100) is low, and its performance for C_2+_ products is still unsatisfactory. The C_2+_ FE of CO_2_RR on the Cu cube, which predominantly exposes the (100) facets, has been revealed to remain below 60%.^[^ ^15^ ^]^

Additionally, the catalyst structure affects the local microenvironment, which has great potential to control the reaction rate, selectivity, stability, etc.^[^ ^16^ ^]^ In particular, the catalyst with the porous channel structure provides a confined space for the reaction, which can tune the diffusion behavior of the reactive/non‐reactive species and influence the accessibility of the reactant to the active site.^[^ ^17^, ^18^ ^]^ For example, He et al. reported that the porous channel structure can increase the surface OH^−^ concentration, enrich the reactant, and thus promote catalytic performance.^[^ ^19^ ^]^ Lang et al. constructed CuIn aerogel with the nanoporous structure to confine the OH^−^ diffusion, resulting in a high local pH microenvironment for efficient acidic CO_2_RR to CO.^[^ ^20^ ^]^ Meanwhile, the porous structure is conducive to exposing more defect sites on the catalyst surface, which helps to boost the *CO coverage for further C–C coupling to produce C_2+_ products.^[^ ^21^, ^22^, ^23^ ^]^

Therefore, the rational design of Cu‐based catalysts—synergistically integrating a porous architecture and abundant Cu (100) facets—thus simultaneously enriching defect sites for *CO retention, establishing a localized alkaline microenvironment and square Cu(100) sites for C_2+_ products formation, represents a promising strategy to overcome the intrinsic limitations of acidic CO_2_RR systems. Here, we developed a porous copper nanosheet (pCu NS) with abundant Cu(100) for efficient acidic CO_2_RR to C_2+_ products in membrane electrode assembly (MEA) configurations. The pCu NS catalyst demonstrated exceptional performance, achieving a record C_2+_ products Faradaic efficiency of 75.01% at 300 mA cm⁻^2^ under a cell voltage of −3.84 V, while providing a CO_2_ single‐pass conversion efficiency up to 74.38%, surpassing conventional Cu catalysts in acidic media. Mechanistic investigations using in‐situ Raman spectroscopy and density functional theory (DFT) calculations confirmed that the porous structure helps to enrich OH⁻ concentration, and the edge sites facilitate *CO intermediate stabilization and promote the hydrogenation of *CO on Cu(100) for C_2+_ production. This work establishes a structural and crystalline orientation‐guided paradigm for acidic CO_2_RR toward the electrosynthesis of multi‐carbon products with enhanced carbon utilization efficiency.

## 2 Results and Discussion

### 2.1 Construction and Characterization

**Figure 1:**
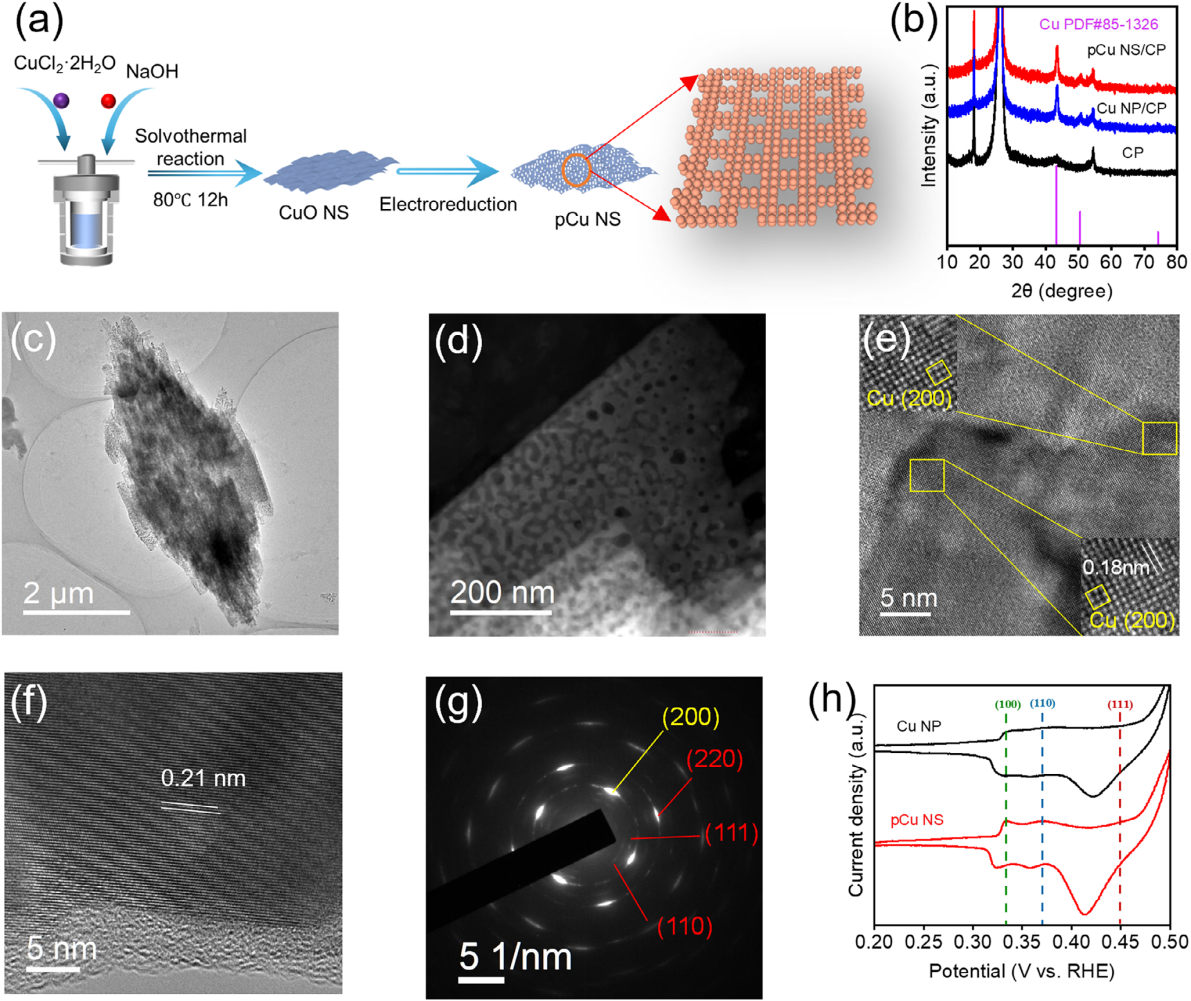
a) Schematic illustration of the preparation procedure of pCu NS. b) XRD pattern of pCu NS and Cu NP. c) TEM images of pCu NS. d) HAADF‐STEM image of pCu NS. e) HRTEM image of pCu NS. f) HRTEM images of Cu NP. g) Selected area electron diffraction (SAED) pattern of pCu NS. h) OH^−^ adsorption tests of pCu NS and Cu NP.

The pCu NS were fabricated through electrochemical reduction of CuO NS (**Figure** **1a**). The CuO NS was obtained via a hydrothermal reaction. Similarly, Cu nanoparticle (Cu NP) obtained from the electroreduction of commercial CuO NP was prepared for comparison. The scanning transmission electron microscopy (SEM) images confirm the smooth surface of CuO NS (Figure S1a‐c, Supporting Information). X‐ray diffraction (XRD) patterns of CuO NS and CuO NP match well with the CuO phase (PDF#48‐1548) (Figure S2, Supporting Information). XPS spectra of CuO NS and CuO NP prove that their Cu valence states are +2 (Figure S3, Supporting Information). The pCu NS and Cu NP were obtained after electroreduction of CuO NS and CuO NP, respectively, at ‐0.3 V versus RHE in CO_2_‐saturated KHCO_3_ electrolyte for ca. 60 min (Figure S4, Supporting Information). XRD patterns prove the successful transformation of CuO to metallic Cu (PDF#85‐1326) after the electrochemical reduction process (Figure 1b). The pCu NS and Cu NP retain the original nanosheet structure like CuO NS and CuO NP, although the surface becomes rough (Figure 1c,f; Figures S1 and S5, Supporting Information). Intriguingly, as characterized by high‐angle annular dark‐field scanning transmission electron microscopy (HAADF‐STEM) and TEM, the pCu NS exhibits a morphology of porous nanosheets, which have numerous pores and pits on each nanosheet, promoting the exposure of low‐coordinated Cu defect sites (Figure 1d; Figures S6 and S7, Supporting Information). Meanwhile, the pore size and Brunauer‐Emmett‐Teller (BET) specific surface area of the catalysts were investigated by N_2_ adsorption–desorption isotherms, the catalyst pCu NS (10.7108 m^2^ g^−1^) has a larger BET surface area compared to Cu NP (6.1964 m^2^ g^−1^) and gives abundant pores of about 4.1 nm (Figure S8, Supporting Information). Additionally, abundant lattice fingers of Cu(200) can be observed in pCu NS, indicating its Cu(100) facet preference, which is different from the CuO NP‐derived Cu with mainly Cu(111) facet preference (Figure 1e,f; Figures S9 and S10, Supporting Information).^[^ ^24^ ^]^ Selective area electron diffraction (SAED) patterns show that the pCu NS exhibit more pronounced Cu(200) crystal plane features than Cu NP, which is consistent with the results of High‐resolution TEM (HRTEM) (Figure 1g; Figure S11, Supporting Information). The surface features of pCu NS and Cu NP were further examined via distinct adsorption behaviors of OH^−^ on different facets of Cu.^[^ ^25^, ^26^ ^]^ It has previously been reported that the anodic oxidation of a Cu surface is preceded by the electrosorption of oxygen, with different Cu facets exhibiting distinctive, reversible adsorption/desorption peaks in the voltammograms.^[^ ^27^, ^28^ ^]^ This has enabled the observation of the surface structure of the Cu by means of a comparison of their oxygen electrosorption patterns, with the peaks at 0.33 V, 0.37 V, and 0.44 V corresponding to hydroxyl electrosorption on Cu(100), Cu(110), and Cu(111) facets, respectively. Notably, the pCu NS catalyst exhibits a dominant (100) facet exposure compared to Cu NP, as evidenced by the enhanced intensity of the characteristic peak at 0.33 V.^[^ ^29^ ^]^ Cu(100) has been widely recognized as the highly active site for the formation of C_2+_ products.^[^ ^14^, ^30^, ^31^, ^32^ ^]^

**Figure 2:**
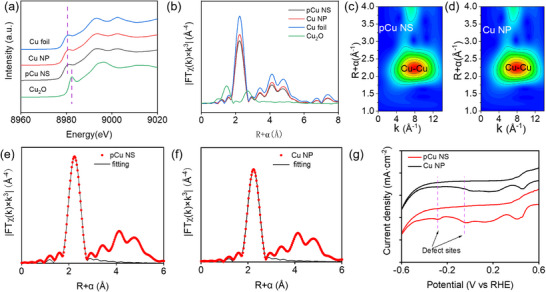
a) Cu K‐edge Normalized XANES spectra of pCu NS, Cu NP, Cu_2_O, and Cu‐foil standard. b) Cu K‐edge Fourier transform (FT) EXAFS spectra of pCu NS, Cu NP, Cu_2_O, and Cu‐foil standard. c‐d) Cu K‐edge wavelet transforms EXAFS 2D plots of pCu NS and Cu NP. e‐f) EXAFS fitting curves in R space of pCu NS and Cu NP. g) Cyclic voltammograms in Ar‐saturated 0.1 M KHCO_3_ aqueous solution of pCu NS.

To further gain insight into the chemical state and local structure of the as‐synthesized sample, X‐ray absorption spectroscopy (XAS) was performed. The Cu K‐edge X‐ray absorption near edge spectra (XANES) confirm that pCu NS and Cu NP are metallic Cu (**Figure** **2a**). The extended Cu K‐edge X‐ray absorption fine structure (EXAFS) of the two samples presents Cu−Cu coordination at 2.23 Å, which is identical to that of Cu foil (Figure 2b). The wavelet transform (WT)‐EXAFS Cu K‐edge spectra also showed the dominant Cu–Cu peak, confirming the metallic state of the catalysts (Figure 2c‐d; Figure S12, Supporting Information). By quantitative EXAFS curve fitting analysis, the coordination number of Cu atom for pCu NS is confirmed to be 8.0, smaller than those of Cu NP (8.8) and Cu foil (12.0), which confirmed that pCu NS exposes more low coordination defect sites (Figure 2e‐f; Figures S13‐S14 and Table S1, Supporting Information). Cyclic voltammetry (CV) tests of pCu NS and Cu NP were conducted in an Ar‐saturated 0.1 M KHCO_3_ aqueous solution to verify further the existence of low‐coordinated defect sites on pCu NS (Figure 2g). The reduction peaks at 0.5 and 0.2 V originated from Cu^2+^ → Cu^+^ and Cu^+^ → Cu, respectively. The peaks at ‐0.3 and ‐0.1 V are attributed to the reduction of oxidized Cu defect sites, with more apparent peaks of pCu NS strongly implying that pCu NS gives more abundant defect sites.^[^ ^33^, ^34^ ^]^ All the above results confirm the successful preparation of pCu NS with abundant nanopores, low coordination defect sites, and Cu(100) facets.

### 2.2 CO_2_RR Performances in the MEA Electrolyzer

**Figure 3:**
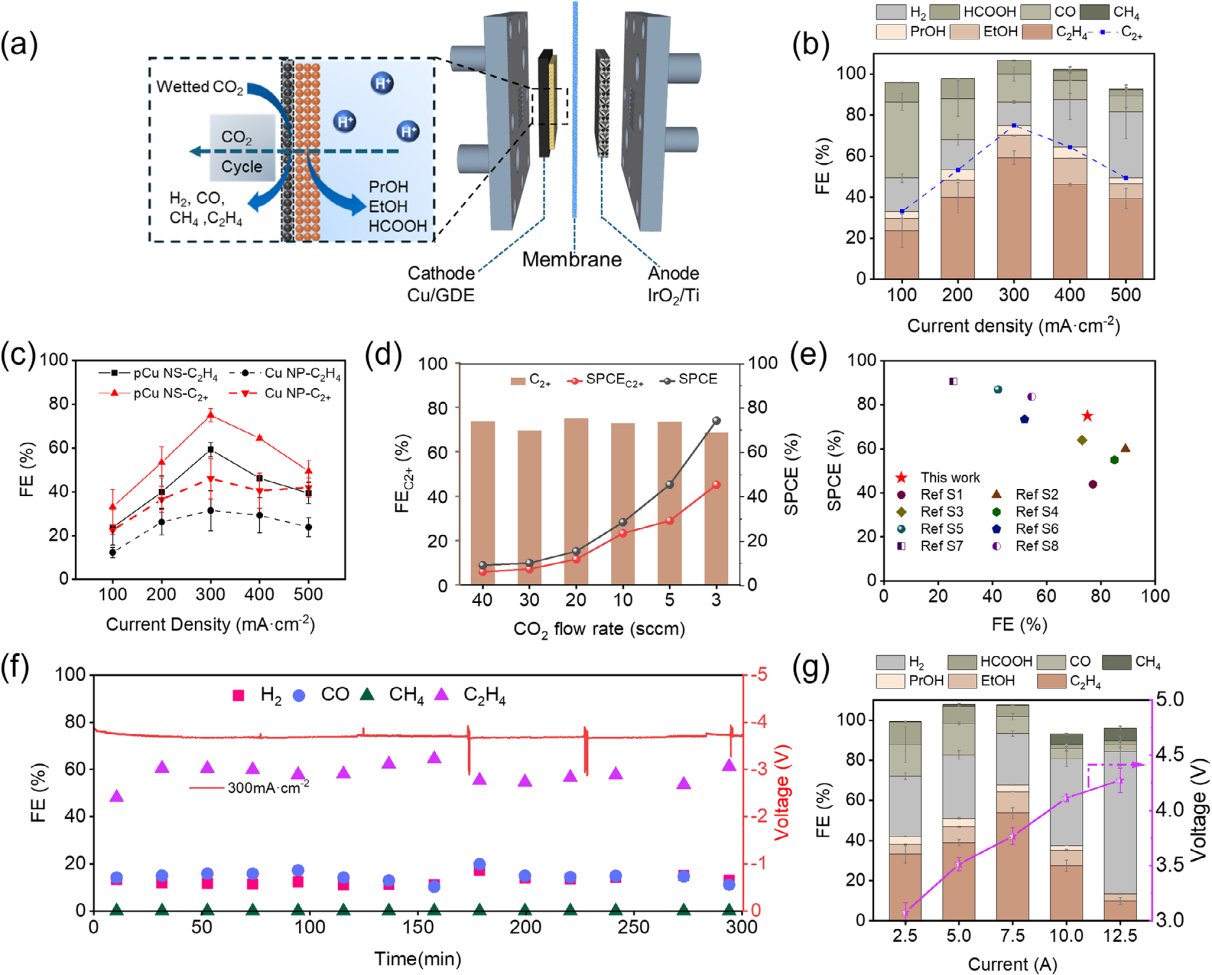
The CO_2_RR performance in the MEA. a) Schematic illustration of the MEA system. b) FEs under different current densities of pCu NS. c) FEs of C_2_H_4_ and C_2+_ products under different current density. d) FEs and SPCE of C_2+_ products obtained for pCu NS at ‐300 mA cm^−2^ under different gas flow rates. (At 3 sccm CO_2_ flow rate, the current density is ‐200 mA cm^−2^) e) Comparison of FE_C2+_ and SPCE with other reported CO_2_RR in acidic conditions. f) The long‐term stability test in 0.05 M H_2_SO_4_ + 0.4 M K_2_SO_4_ at ‐300 mA cm^−2^ on pCu NS. g) Current‐dependent FE (left) and cell voltage (right) of pCu NS in 25 cm^2^ MEA cell with 0.05 M H_2_SO_4_ + 0.4 M K_2_SO_4_ anolyte. (The error bars represent the standard deviation (SD) of three replicates, with data presented as mean ± SD).

We assessed the CO_2_RR performance of pCu NS and Cu NP in the current density range from 100 to 500 mA cm^−2^ using a MEA electrolyzer setup (**Figure** **3**a; Figure S15, Supporting Information). The 4 cm^2^ iridium oxide‐based Ti‐mesh (IrO_2_/Ti) was used as an anode electrode for the acidic oxygen evolution reaction. The electrodes were separated by a proton‐exchange membrane (PEM, Nafion N212), and 0.05 M H_2_SO_4_ + 0.4 M K_2_SO_4_ (pH = 2) served as the anolyte. Online gas chromatography (GC) and headspace GC spectroscopy (HS‐GC) were employed to analyze gas products and liquid products, respectively (Figure S16, Supporting Information). The liquid products were collected using a cold trap connected to the cathode gas outlet. The CO_2_RR activities of pCu NS and Cu NP were first examined by linear sweep voltammetry (LSV). Figure S17 (Supporting Information) shows that Cu catalysts preferably engender CO_2_RR rather than HER, and pCu NS has a lower operating voltage at the same operating current density, which proves that pCu has higher electrocatalytic activity. Figure 3b and Figure S18 (Supporting Information) display the FEs of different CO_2_RR products on pCu NS and Cu NPg at the current density range from 100 to 500 mA cm^−2^. Compared with Cu NP, pCu NS gives a lower voltage at the same current density (Figure S19, Supporting Information). At a cell voltage of 3.84 V, the FE of C_2+_ products over pCu NS could reach 75.01% with a partial current density of 225.03 mA cm^−2^, and additionally, the FE of C_2_H_4_ products is as high as 59.34% (Figure 3c; Figure S20, Supporting Information). At the same time, the FEs of H_2_ and C_1_ were suppressed to 12.2 and 20.21%, respectively. When the current density exceeds 400mA cm^−2^, the competitive HER increases sharply, and CH_4_ appears. In comparison, the maximum C_2+_ FE for the Cu NP catalyst was only 45.3%, and larger amounts of H_2_ and C_1_ products were produced with FEs of 24.5 and 24.1%, respectively (Figure S18, Supporting Information). Besides, we further evaluate the electrochemically active surface area (ECSA) from double‐layer capacitance values (C_dl_) determined by CV measurement at different scan rates in the non‐Faradaic region (Figure S21, Supporting Information). As shown in Figure S22 (Supporting Information), the ECSA of pCu NS (1.08 mF cm^−2^) is similar to Cu NP (0.93 mF cm^−2^), excluding the effect of ECSA on the catalytic activity.

Acidic CO_2_RR is advantageous in overcoming carbon utilization limitations observed in neutral and alkaline solutions. Upon reducing the flow rate of CO_2_ from 40 to 3 standard cubic centimeters per minute (sccm), the SPCE for all the products over pCu NS increased from 6.26% to 74.38%, and the SPCE for C_2+_ products could reach 45.5% at 3 sccm (Figure 3d). The SPCE and FE of C_2+_ products on pCu NS are comparable to those of state‐of‐the‐art Cu‐based electrocatalysts in acidic electrolyte (Figure 3e; Table S2, Supporting Information). Additionally, long‐term CO_2_RR tests were performed to evaluate the durability of pCu NS. The long‐term electrocatalysis performance of the pCu NS catalyst was performed at 300mA cm^−2^. As shown in Figure 3f, both the potential and the FE of C_2_H_4_ remained stable during the entire electrolysis period, suggesting that the pCu NS catalyst with rich defect sites was stable at a high current density for long‐term electrolysis to produce C_2+_ products. Meanwhile, the XRD spectra (Figure S23, Supporting Information) and SEM images (Figure S24, Supporting Information) of pCu NS after CO_2_RR are similar to that of as‐prepared pCu NS, demonstrating the stable structure of pCu NS for CO_2_RR. Additionally, the post‐stability CV curve of pCu NS further confirmed the structural stability of the catalyst, with the Cu defect sites remaining preserved (Figure S25, Supporting Information). To further verify the potential for large‐scale application, a larger MEA configuration with an area of 25 cm^2^ was adopted to understand the activity of pCu NS. At a large current of 7.5 A, the FE of 67.7% for C_2+_ products was obtained (Figure 3g). Considering the experimental observations mentioned above, the defect‐rich nanoporous pCu NS with abundant (100) crystal facets can inhibit the production of H_2_ and C_1_ and improve the selectivity of C_2+_ products in acidic electrolytes.

### 2.3 In Situ Raman Characterization

**Figure 4:**
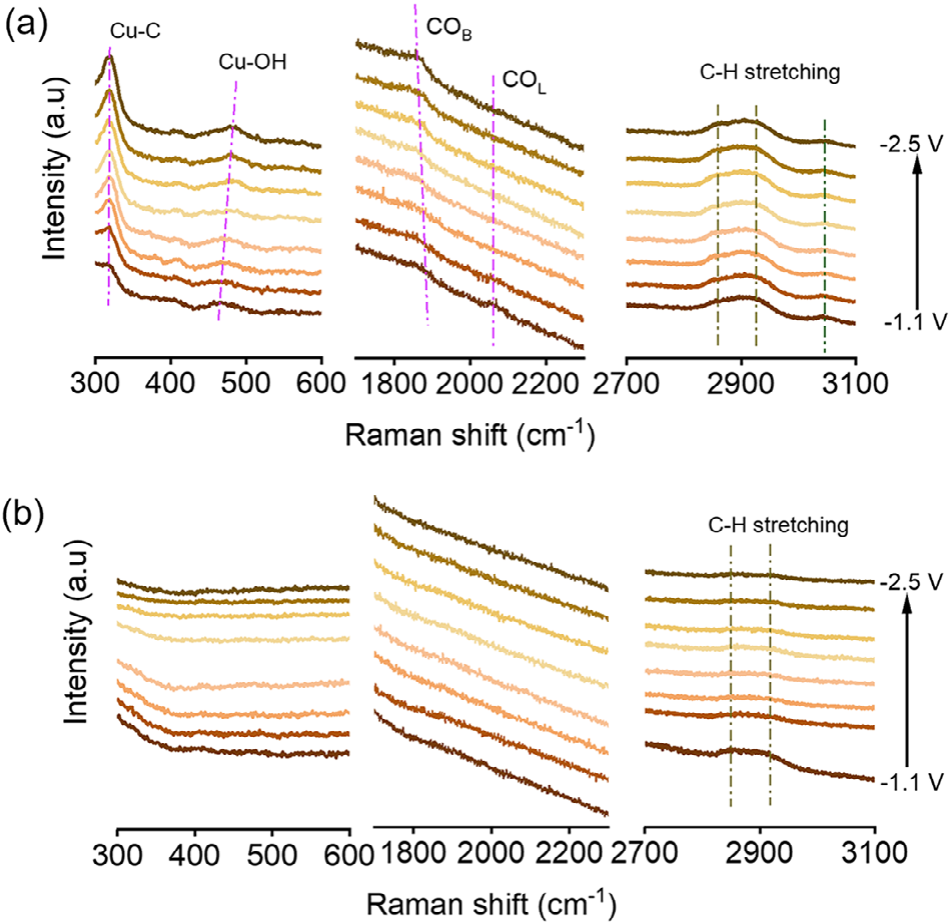
In situ Raman spectra for a) pCu NS and b) Cu NP at various potentials (versus Ag/AgCl) during CO_2_RR in acidic electrolyte (0.05 M H_2_SO_4_ + 0.4 M K_2_SO_4_).

To understand the mechanism of the reduction of CO_2_RR on pCu NS in acidic electrolytes, in‐situ Raman spectroscopy measurements were employed to determine the key reaction intermediates over the pCu NS and Cu NP catalysts. As depicted in **Figure** **4a**, the Raman band located at 320 cm^−1^, 1850–1900 cm^−1^, and 2069–2080 cm^−1^ appeared on the pCu NS catalyst, which was attributed to frustrated rotation and stretching vibrations of Cu‐CO bonds, bridged (CO_B_) and linear (CO_L_) adsorption configurations of *CO, respectively.^[^ ^35^, ^36^ ^]^ However, no apparent band of Cu‐CO stretching and *CO emerged over Cu NP, indicating stronger adsorption and the high coverage of *CO species on the defect‐rich pCu NS catalyst (Figure 4b). Therefore, compared with Cu NP, pCu NS exhibits a higher *CO coverage, affording sufficient *CO for C–C coupling to C_2+_ products. Meanwhile, an obvious band at ≈488 cm^−1^ appeared on pCu NS. This signal is typically attributed to Cu‐OH species,^[^ ^37^, ^38^ ^]^ corresponding to the fact that the nanoporous structure helps to create a locally alkaline environment on the surface of pCu NS in the acidic electrolyte. The local alkaline environment is conducive to inhibiting HER for upgrading CO_2_RR. In addition, the Raman bands at 2834–2960 cm^−1^ were observed on pCu NS, which can be attributed to the C‐H vibrational mode of *CO‐COH, *CO‐CHO, *CH_x_, etc. However, the C‐H vibration is faintly on Cu NP, further confirming the superior performance of pCu NS for CO_2_ reduction to C_2+_ products such as C_2_H_4_.

### 2.4 Mechanistic Studies

**Figure 5:**
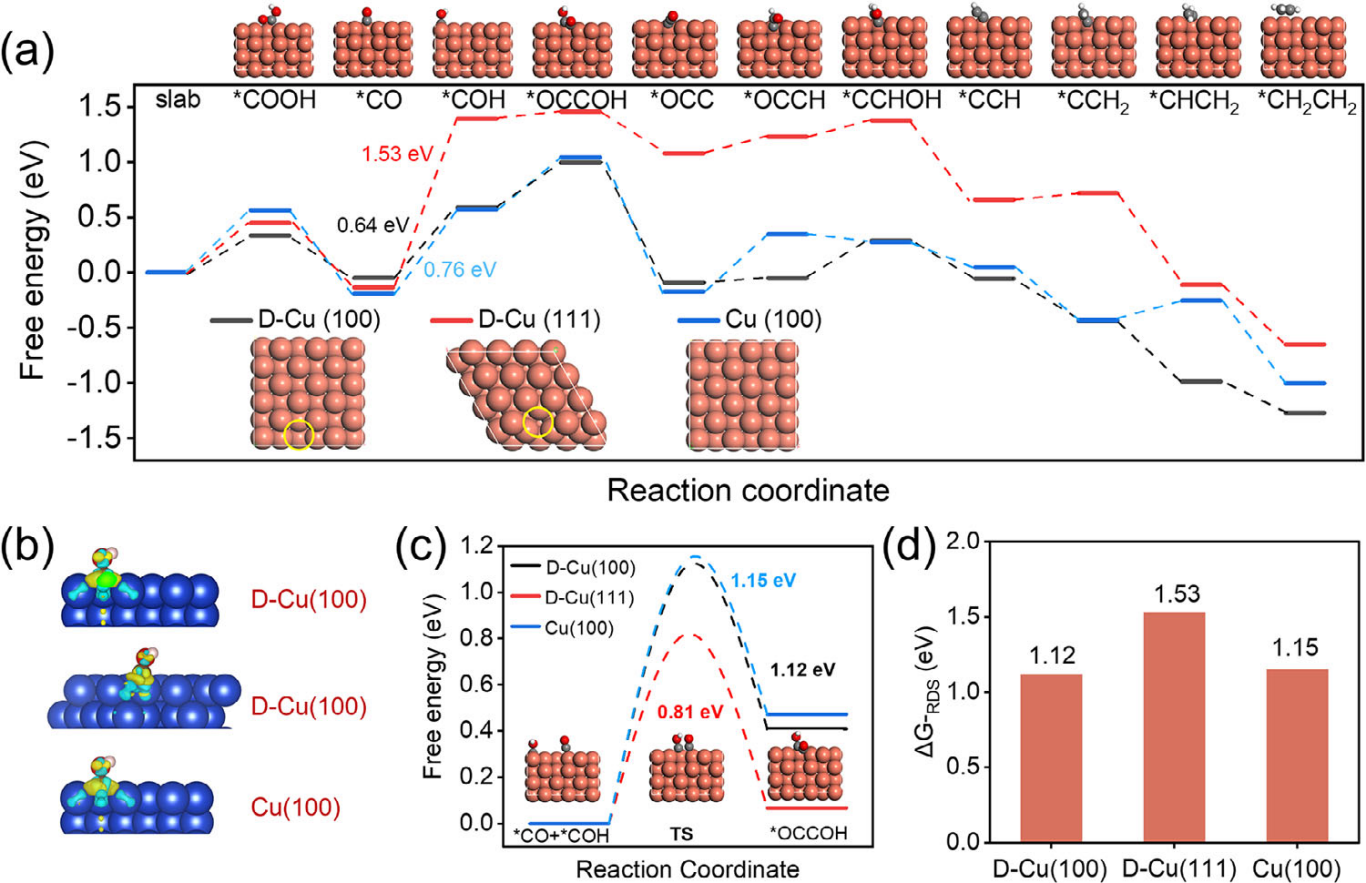
a) DFT‐calculated Gibbs free energy of the intermediates on D‐Cu(100) **(black)**, D‐Cu(111) **(red),** and Cu(100) **(blue)** surfaces. b) Visualized differential charge density during *CO protonation (*CO + *H →*COH). c) The reaction free energy barrier of the *CO+*COH→*OCCOH pathways on D‐Cu(100), D‐Cu(111) and Cu(100) surfaces. d) The Gibbs Energy Barrier of rate‐determining steps (RDS) during CO_2_RR of D‐Cu(100), D‐Cu(111), and Cu(100) surfaces. D‐Cu(100) and D‐Cu(111) represents Defect‐Cu(100) and Defect‐Cu(111), respectively.

Density functional theory (DFT) calculations were conducted to elucidate the reaction mechanism for high CO_2_RR activity to afford C_2+_ products over pCu NS. The reaction pathway for ethylene (C_2_H_4_) formation was systematically investigated as a representative. Three distinct models—Defect Cu(100), Defect Cu(111), and Cu(100)—were constructed for investigation (**Figures** **5**a, S26 and Table S3, Supporting Information). As shown in Figure 5a,c, D‐Cu(111) exhibits a lower kinetic energy barrier (0.81 eV) for C─C bond formation (*CO + *COH → *OCCOH) but a higher energy barrier (1.53 eV) for *CO protonation (*CO + *H →*COH), demonstrating the overall reaction is governed by the rate‐determining step (RDS) of *CO hydrogenation on D‐Cu(111). Differential charge density analysis of adsorbed *COH intermediates reveals that D‐Cu(100) and Cu(100) facilitate electron density transfer compared to D‐Cu(111) (Figure 5b), illustrating the weak adsorption of *COH on D‐Cu(111) and the higher Gibers free energy for its formation. Additionally, the higher transition state energy barrier for C─C coupling on D‐Cu(100) (1.12 eV) and Cu(100) (1.15 eV) than the hydrogenation of *CO (0.64 eV and 0.76 eV, respectively) unraveled the production of C_2+_ products was determined by C─C coupling on D‐Cu(100) and Cu(100). The energy barriers of the RDS on D‐Cu(111) (1.53 eV) is larger than that on D‐Cu(100) (1.12 eV) and Cu(100) (1.15 eV), demonstrating that D‐Cu(100) and Cu(100) are more favorable for ethylene production than D‐Cu(111) (Figure 5d). These results highlight the crucial role of Cu(100) in promoting the hydrogenation of *CO for C_2+_ products formation in pCu NS.

## 3 Conclusions

In summary, we have successfully fabricated porous Cu nanosheets (pCu NS) through an in‐situ topological transformation of CuO NS, which features abundant defect sites with preferentially exposed (100) crystal facets. This unique architecture was demonstrated as an efficient electrocatalyst for CO₂ reduction reaction under acidic conditions. Mechanism study revealed that the synergistic combination of nanoporous structure, (100) facets, and defect sites establishes a local alkaline microenvironment, enhances the surface coverage of key CO* intermediates, lowering CO* intermediates protonation barriers, thus finally for upgrading C₂_+_ product formation. We achieve an impressive Faradaic efficiency of 75.01% for C₂_+_ products with a notable partial current density of 225.03 mA cm^−2^, while providing a high CO_2_ single‐pass conversion efficiency up to 74.38% on pCu NS in acidic MEA configuration. This study advances catalyst design principles by elucidating the critical role of crystal facet and defect‐rich nanoporous structure, offering a new paradigm for developing copper‐based electrocatalysts for acidic CO_2_RR.

## Conflict of Interest

The authors declare no conflict of interest.

## Supporting information



Supporting Information

## Data Availability

The data that support the findings of this study are available from the corresponding author upon reasonable request.
