# Supplementary material for: Defect‐Rich Porous Cu with Abundant Cu(100) for Acidic CO2 Electroreduction in Membrane Electrode Assembly
Source: Adv Sci (Weinh). 2025 Jul 26;12(40):e10161. doi: 10.1002/advs.202510161 (PMC12561329; doi:10.1002/advs.202510161)
Supplement: Supplementary file 1 — Supporting Information [file ADVS-12-e10161-s001.docx]

Supporting information

Defect-rich Porous Cu with Abundant Cu(100) for Acidic CO_2_ Electroreduction in Membrane Electrode Assembly

Qiang Fang, Yunzhen Jia, Xuelei Lang, Geng Li, Tao Zhao, Dazhong Zhong^*^, Jinping Li and Qiang Zhao^*^

College of Chemistry and Chemical Engineering, Shanxi Key Laboratory of Gas Energy Efficient and Clean Utilization, Taiyuan University of Technology, Taiyuan 030024, Shanxi, P.R. China.

E-mail: [zhaoqiang@tyut.edu.cn](mailto:zhaoqiang@tyut.edu.cn); [zhongdazhong@tyut.edu.cn](mailto:zhongdazhong@tyut.edu.cn)

**Experimental sections**

**1. Materials**

Sodium hydroxide (NaOH), Copric chloride dihydrate (CuCl_2_·2H_2_O), sulfuric acid (H_2_SO_4_) and methanol (CH_3_OH) were purchased from Sinopharm Chemical Reagent Co., Ltd. potassium sulfate (K_2_SO_4_) and potassium bicarbonate (KHCO_3_) were purchased from Aladdin Co. All the chemicals were used as received without further purification. Deionized water with a resistivity of 18.25 MΩ cm^-1^ was used to prepare aqueous solutions.

**2. Characterization**

The Bruker D8 Advance diffractometer with Cu–Kα radiation (1.541874 Å) was used to record the X-ray diffraction (XRD) patterns of the materials under investigation. The Hitachi SU8010 scanning electron microscope (SEM) was utilized to determine the surface micromorphology of the materials. The WSCAL-ab 220i-XL spectrometer (VG Scientific) with Al–Kα radiation was used to perform X-ray photoelectron spectroscopy (XPS). The transmission electron microscope (TEM) and high-resolution TEM (HRTEM) images were obtained using a FEI Tecnai G2 F20. Further, In-situ Raman (Renishaw) spectroscopy (with λ = 785 nm) of the samples was used to further analyze the compositional information. Brunauer–Emmett–Teller (BET) surface areas were measured with a Micromeritics TriStar II 3020 instrument based on N_2_ adsorption-desorption isotherms recorded at 77 K.

**3. Syntheses of porous Cu nanosheets (pCu NS) and Cu nanoparticles (Cu NP).**

Copper oxide nanosheets (CuO NS) were synthesized following a previously reported procedure with several modifications. Initially, 2.131 g of CuCl_2_·2H_2_O were dissolved in 100 mL of water and vigorously stirred for 30 min to ensure the formation of a uniform Cu^2+^ solution. Subsequently, 20 mL Cu^2+^ solution was withdrawn and slowly added dropwise to 50 mL of 3M NaOH under intense stirring. The mixture was rapidly heated to 50 °C and maintained at this temperature for 1 hour, with continuous vigorous stirring. Following this step, the solution was transferred into a Teflon-lined autoclave, sealed tightly, and heated at 80 °C for an additional 12 hours. After the reaction, the system was allowed to cool naturally to room temperature. The resulting precipitate was collected by centrifugation and thoroughly rinsed with distilled water and ethanol several times to remove any residual alkaline salts. The precipitate was then dried in a vacuum oven at 60 °C for 12 hours to obtain the final product.

To prepare the pCu NS, a constant voltage topological reduction process was employed at −0.9V versus Ag/AgCl for 1 hour in a CO_2_-saturated 0.1M KHCO_3_ solution.

**4. Preparation of electrodes.**

**Cathode electrodes:** Typically, 9.68mg of CuO NS and 1mL of methanol were mixed by sonicating for 60min, and 60μL of Nafion solution was added, followed by sonicating for another 60min to obtain a homogeneous catalyst ink. The catalyst ink was then sprayed on hydrophobic porous polytetrafluoroethylene gas diffusion layer (GDE, 2.2 cm×2.2 cm). The GDE before and after loading catalysts was weighed to determine the loading amount of the catalyst (~1.6 mg cm^−2^). The pCu NS electrode was obtained via in situ electrochemical reduction from CuO NS electrode for 60min in CO_2_-saturated 0.1M KHCO_3_, with constant voltage conditions at -0.3 V vs RHE. The electrode of Cu NP was obtained in the same method.

**Anode electrodes:** Titanium-mesh-supported iridium oxide (IrO_x_/Ti-mesh, 1 mg·cm^-2^) was used as the anode electrode.

**5. Electrochemical measurements**

All CO_2_RR experiments were performed using an MEA electrolyser with an active area of 4 cm^2^ and the electrochemical data were collected using the Princeton Electrochemical Workstation (PARSTAT MC). The cathodic compartment and anodic compartment were separated by Nafion 212 membrane with catalysts to be measured as the cathode, and IrO_x_/Ti-mesh as the anode electrode. During a CO_2_RR experiment, the aqueous 0.4 M K_2_SO_4_ +0.05M H_2_SO_4_ anolyte was circulated through the anode flow channel using a Gas-liquid mixed flow pump. The CO_2_ gas flow rate, supplied at a rate of 50 standard cubic centimeters per minute (sccm), was bubbled through water for humidification prior to entering the electrolyser. All voltages reported are full cell voltages without iR compensation.

**6. CO_2_RR product analysis**

Unless otherwise stated, CO_2_ gas was let into gas chamber of MEA electrolyser at ambient pressure and room temperature and then injected into a gas chromatograph (GC; Agilent GC 8890) after CO_2_RR to analyze gas products. The GC was equipped with a thermal conductivity detector (TCD) for analyzing H_2_, and a flame ionization detector (FID) for analyzing carbonaceous substances, while calibrated by using standard mixture gas before measurements. Each quantitative sampling was performed three times to achieve accurate results. The Faradaic efficiency of the products was calculated using the following formula:

**Gas products:** $\boldsymbol{FE}_{\boldsymbol{gas}}\mathbf{=}\frac{\boldsymbol{nFVx}}{\boldsymbol{I}}\boldsymbol{\times100\%}$

Where *n* is the number of electrons transferred, *F* is Faraday constant (96485 C mol^-1^), *V* is the total molar flow rate of gas, *x* is the mole fraction of the product, and I is the total current.

**Liquid products:**${FE}_{liquid}=\frac{nFy}{Q}\times100\%$

Where *n* is the number of electrons transferred, *F* is Faraday constant (96485 C mol^-1^), *y* is the is the molar of the product, and *Q* is the total amount of charge passed through the electrode.

**Single-pass conversion efficiency (SPCE):**

$$SPCE=\frac{60s\times\sum(j\times x_{i}\times{FE}_{i}\div(N_{i}\times F))}{flow rate (L/min)\times1 min\div24.05 (L/min)}\times100\%$$

Where $j$ is current density, $x_{i}$ is mole ratio of CO_2_ to *i* product, ${FE}_{i}$ is the faradaic efficiency of *i* product, $N_{i}$ is thenumber ofelectron transfer for *i* product molecule.

**7.** **In-situ Raman spectra tests**

In situ Raman spectroscopy measurements were performed using a Renishaw inVia spectrometer coupled with a customized electrochemical cell. The electrolyte is the 0.05 M H_2_SO_4_ + 0.4 M K_2_SO_4_ (pH = 2) aqueous solution, and a Nafion N212 cation exchange membrane was employed to separate the anode/cathode compartments. A three-electrode configuration was employed, with a carbon paper-supported catalyst as the working electrode, a carbon rod as the counter electrode, and Ag/AgCl as the reference electrode. Spectra were acquired under constant potential application (5 min equilibration time) at 0.2 V intervals across the potential range of −1.1 to −2.5 V (vs. Ag/AgCl), using an excitation wavelength of 785 nm.

**8. Activation and Preservation of the cation exchange membrane.**

The standardized activation protocol for Nafion N212 cation exchange membranes involves sequential boiling treatments: initial immersion in 5 wt% H_2_O_2_ at 80°C for organic contaminant removal, followed by treatment in 0.5 M H_2_SO_4_ at 80°C for complete protonation (H⁺ form conversion) and cation impurity elimination. After thorough rinsing with deionized water, the membrane is hydrated in 80°C ultrapure water to establish equilibrium water uptake and optimal proton conductivity. Activated membranes were immersed in ultrapure water until immediate use to prevent dehydration-induced performance degradation. Each of these steps should be maintained for one hour.

**9. DFT calculations**

*Vienna ab initio simulation package* (VASP) was used to calculate period *density functional theory* (DFT) and spin polarization. The *Perdew-Burke-Ernzerhof generalized approximation* (GGA-PBE) was used to handle exchange correlations, and 450 eV was chosen as the cut-off energy. The self-consistency of electrons was assessed using an energy threshold of 10^-5^ and an ionic step convergence criterion of 0.02 eV/ Å. The Van der Waals (vdW) effect was considered by using Grimmer’s DFT-D3 correction method to correct the dispersion effect. To determine precise lattice parameters, a 7×7×7 K-points mesh was employed for lattice optimization of the Cu unit cell. Materials Studio and VESTA^[1]^ software was utilized to generate D-Cu(100), D-Cu(111) and Cu(100) models together with a 15 Å vacuum layer. VASPKIT^[2]^ used zero-point vibrational energy (ZPE) and frequency analysis to examine the thermodynamic corrections for intermediate adsorption and stability. A 2 × 2 × 1 Monkhorst-Pack K-points grid was also employed to optimize the pure slab and the CO_2_RR adsorption process. To achieve a more realistic simulation of the catalyst surface, we fix two layers at the bottom of the catalyst surface while allowing the surface atoms to relax. The isosurface of charge density was set at 0.001 eV/ Å^3^. The preliminary location of transition states was achieved through climbing-image nudged elastic band (CI-NEB) simulations ^[3]^, with subsequent optimization employing the dimer method ^[4]^. Systematic verification confirmed all identified transition states exhibited singular imaginary vibrational frequencies, thereby validating their structural authenticity.

The Gibbs free energy (∆G) of each oxygen evolution reaction steps is calculated by the following formula:

$$\boldsymbol{\Delta}\boldsymbol{G}\mathbf{=}{\boldsymbol{\Delta}\boldsymbol{E}}_{\boldsymbol{DFT}}\boldsymbol{+\Delta}\boldsymbol{ZPE}\mathbf{-}\boldsymbol{T}\boldsymbol{\Delta}\boldsymbol{S}$$

Where ${\Delta E}_{DFT}$, $\Delta ZPE,$ $\Delta S$are the change of DFT total energy, zero-point energy, and entropy from the initial to the final state.

**10. Statistical Analysis**

The FE results are presented as mean ± standard deviation (SD) of three replicates. Statistical analyses and linear fitting of C_dl_ were performed using Origin 2019b (v9.6).

**
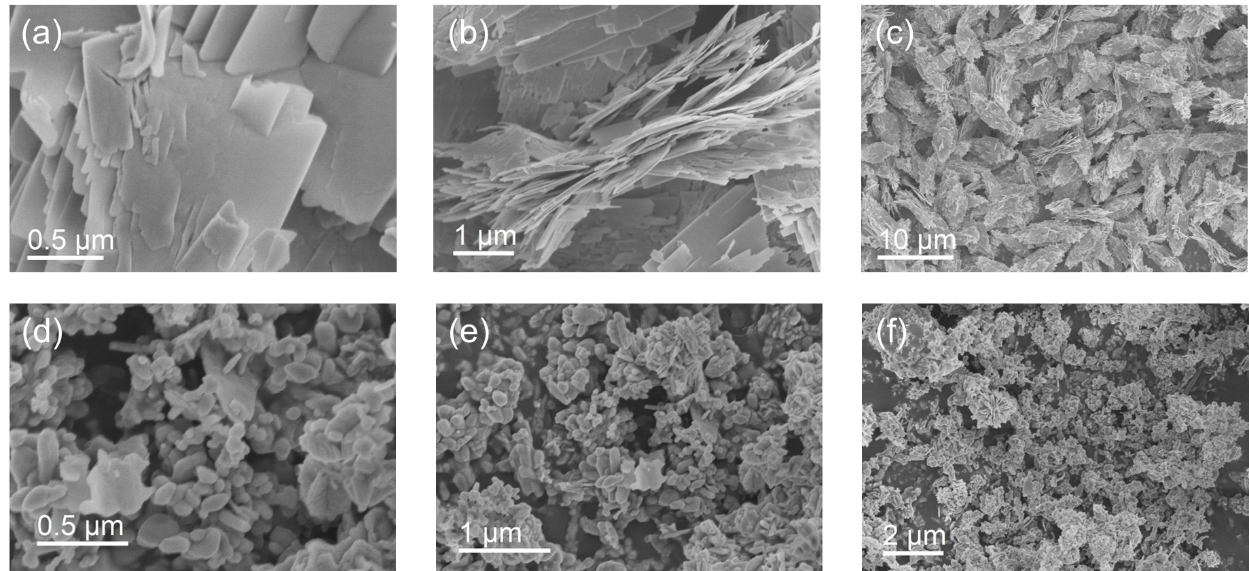
**

**Figure S1.** SEM images of (a-c) CuO NS and (d-f) CuO NP at different magnifications.


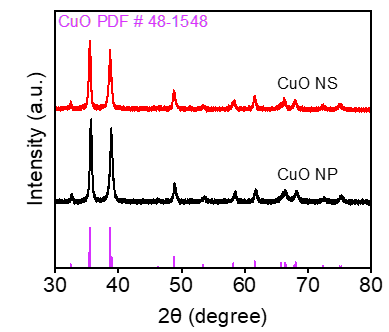


**Figure S2.** XRD patterns of CuO NS and CuO NP.

**
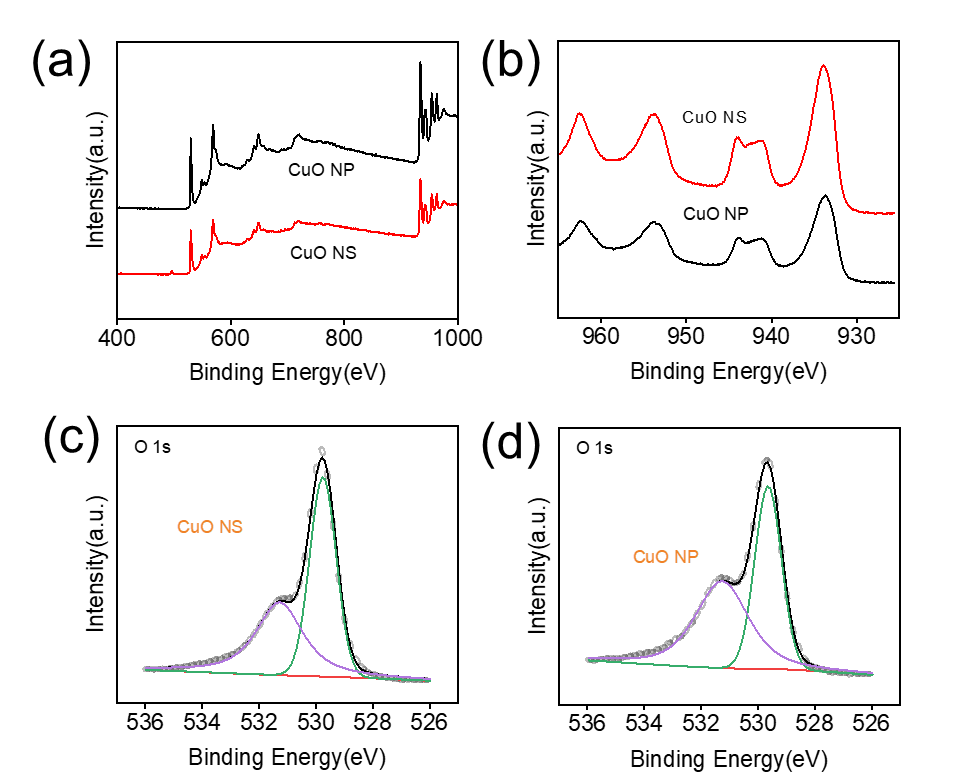
**

**Figure S3.** XPS spectras. (a). XPS survey spectrums of CuO NS and CuO NP (b) Cu 2p spectra of CuO NS and CuO NP (c)O 1s spectra of CuO NS and (d) CuO NP.


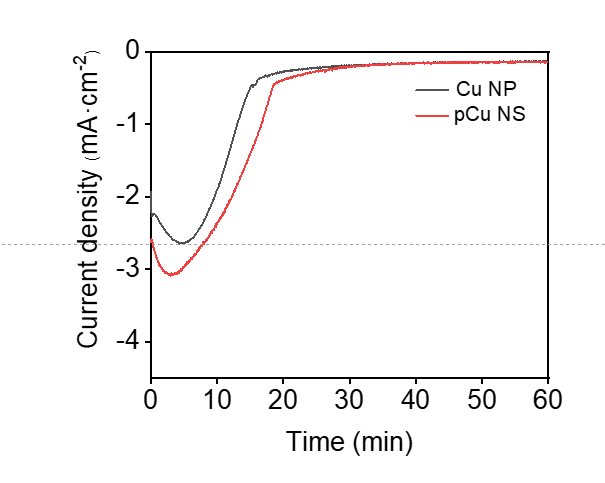


**Figure S4.** Current density curves of constant voltage reduction process of pCu NS and Cu NP.

**
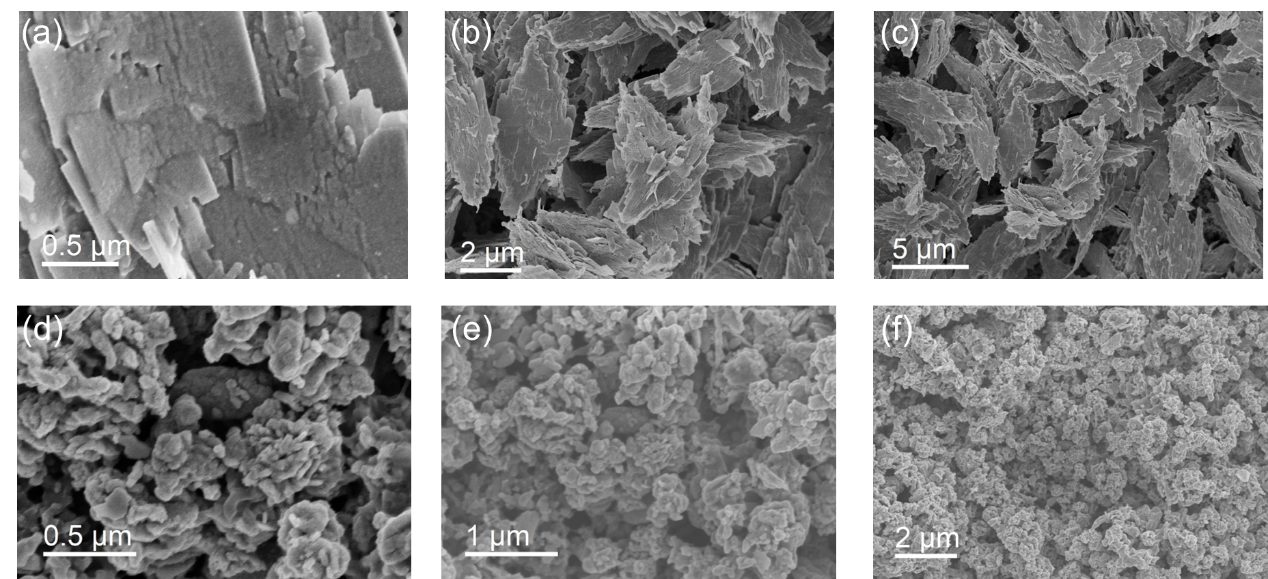
**

**Figure S5.** SEM images of (a-c) pCu NS and (d-f) Cu NP at different magnifications.

**
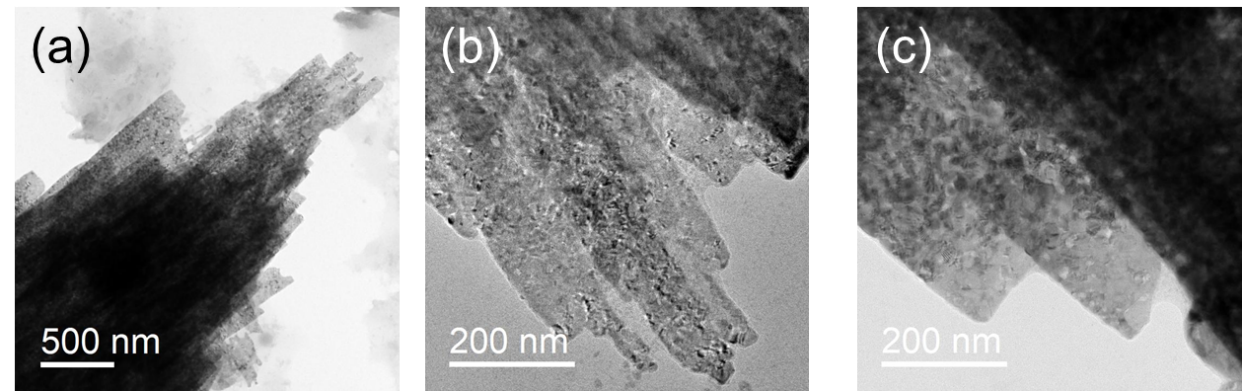
**

**Figure S6.** TEM images of pCu NS at different magnifications.

**
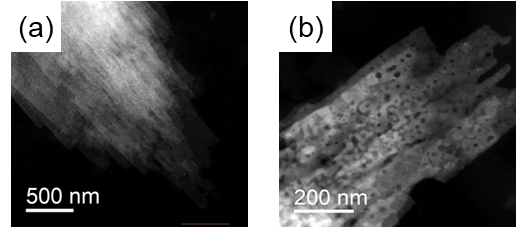
**

**Figure S7.** HAADF-STEM images of pCu NS at different magnifications.

**
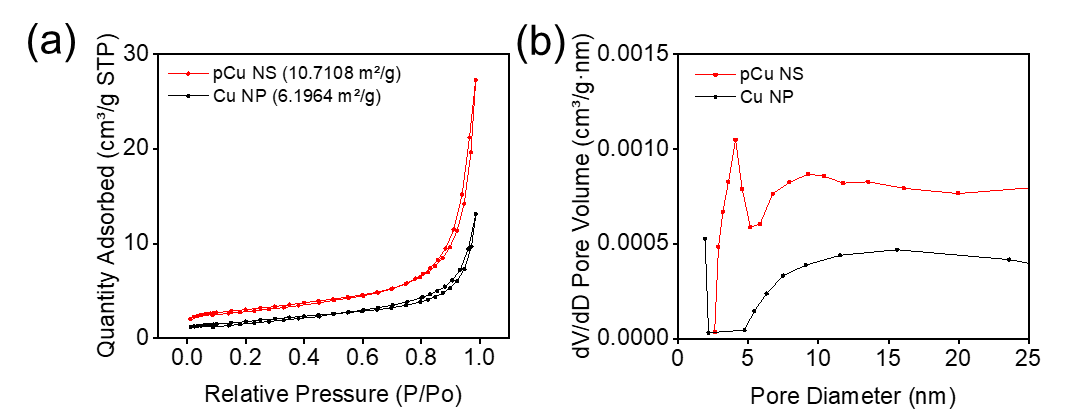
**

**Figure S8.** (a) N_2_ adsorption-desorption isotherms of pCu NS and Cu NP. (b) The pore size distribution of pCu NS and Cu NP.

**Figure S9.** HRTEM images of pCu NS with Cu (100) at different positions.

**Figure S10.** TEM images of Cu NP at different magnifications.

**
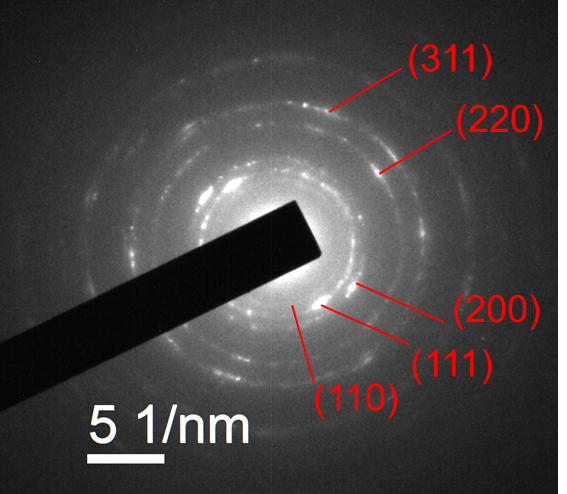
**

**Figure S11**. Selected area electron diffraction (SAED) pattern of Cu NP.

**
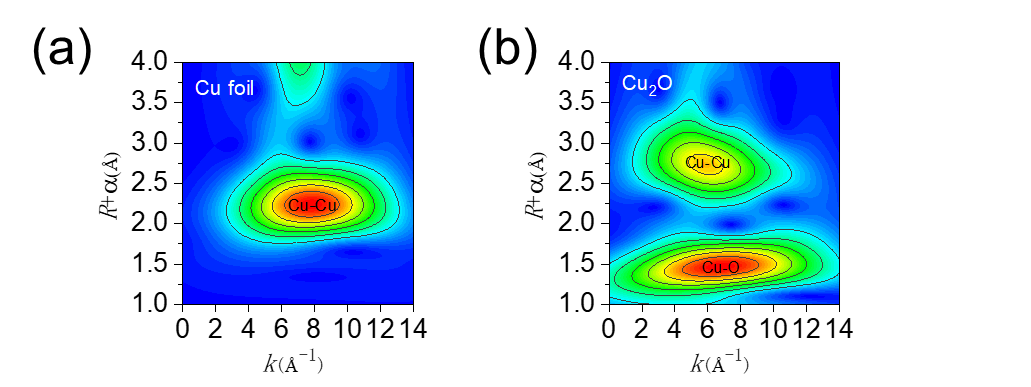
**

**Figure S12.** Cu K-edge wavelet transforms EXAFS 2D plots of Cu-foil (a) and Cu_2_O (b) standard.

**
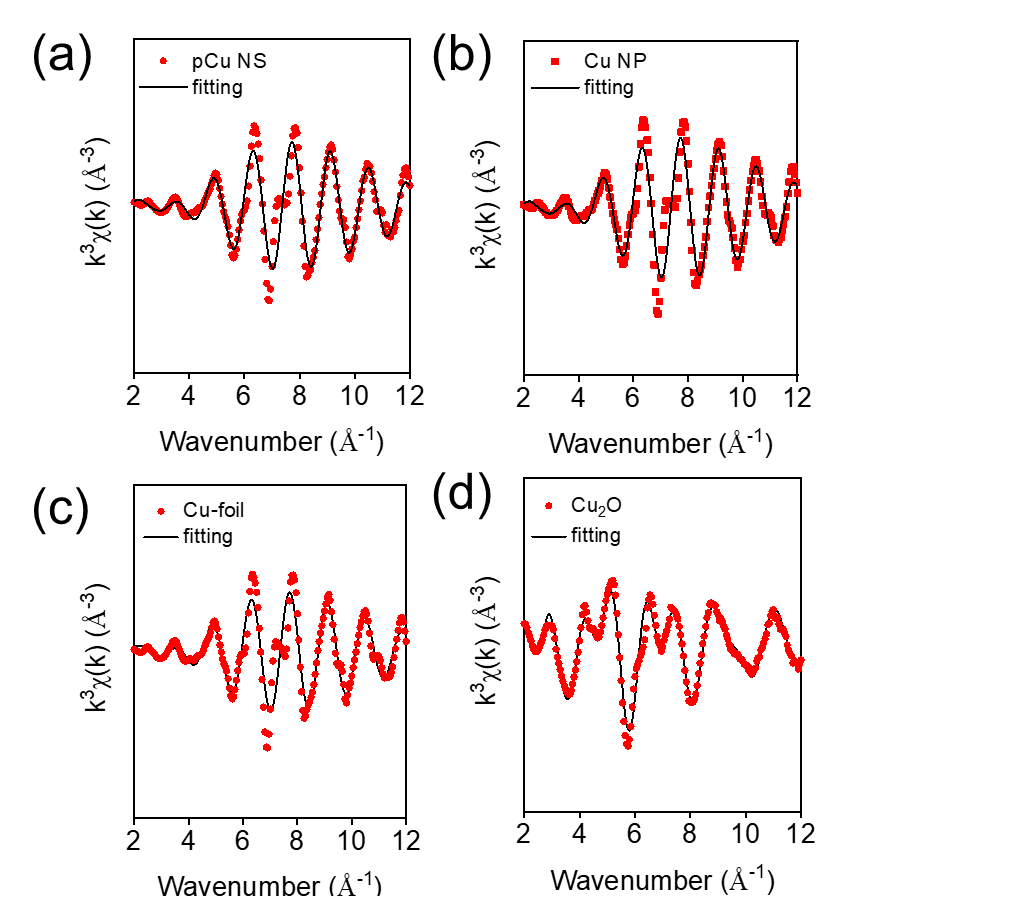
**

**Figure S13.** Cu K-edge EXAFS spectra in k-space for pCu NS (a), Cu NP (b), Cu_2_O(c), and Cu-foil (d) standard.

**
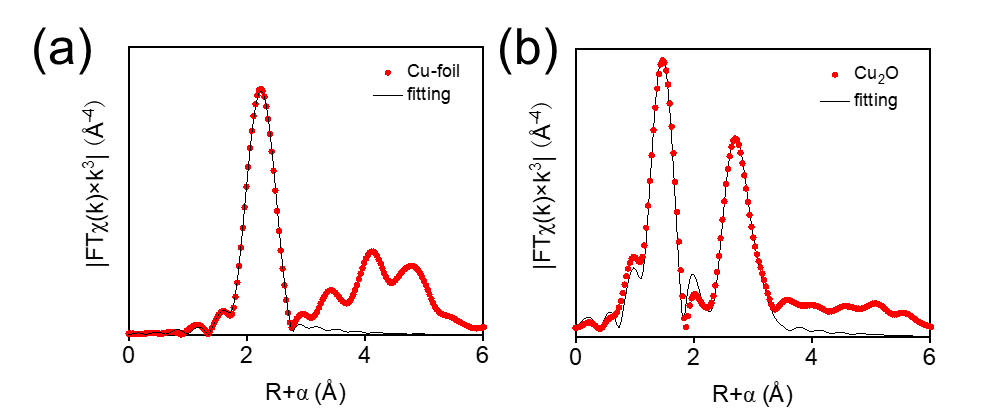
**

**Figure S14.** FT-EXAFS spectra at the Cu K-edge of standard for (a) Cu foil and (c)Cu_2_O

**
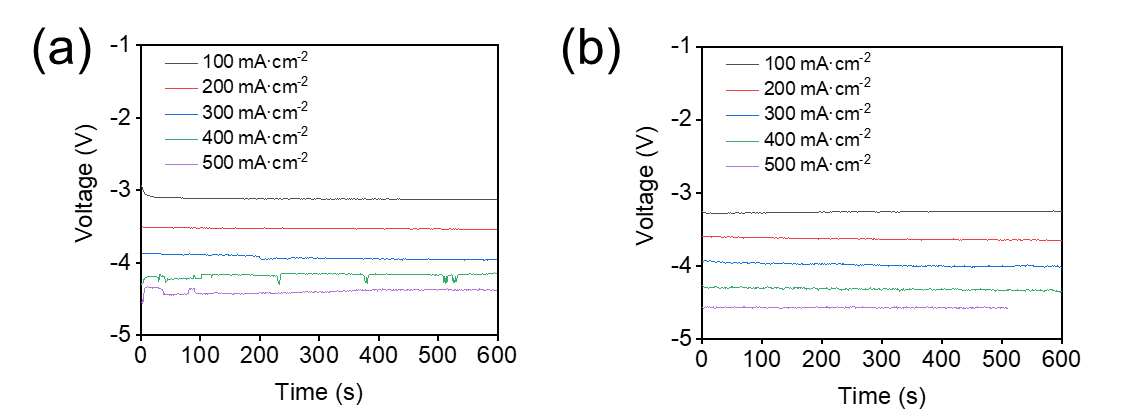
**

**Figure S15.** Voltage curves of gas products of (a) pCu NS and (b) Cu NP during constant current testing.

**
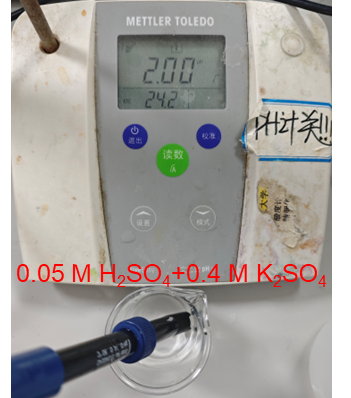
**

**Figure S16.** The pH value of the anode electrolyte (0.05 M H_2_SO_4_+0.4 M K_2_SO_4_).

**
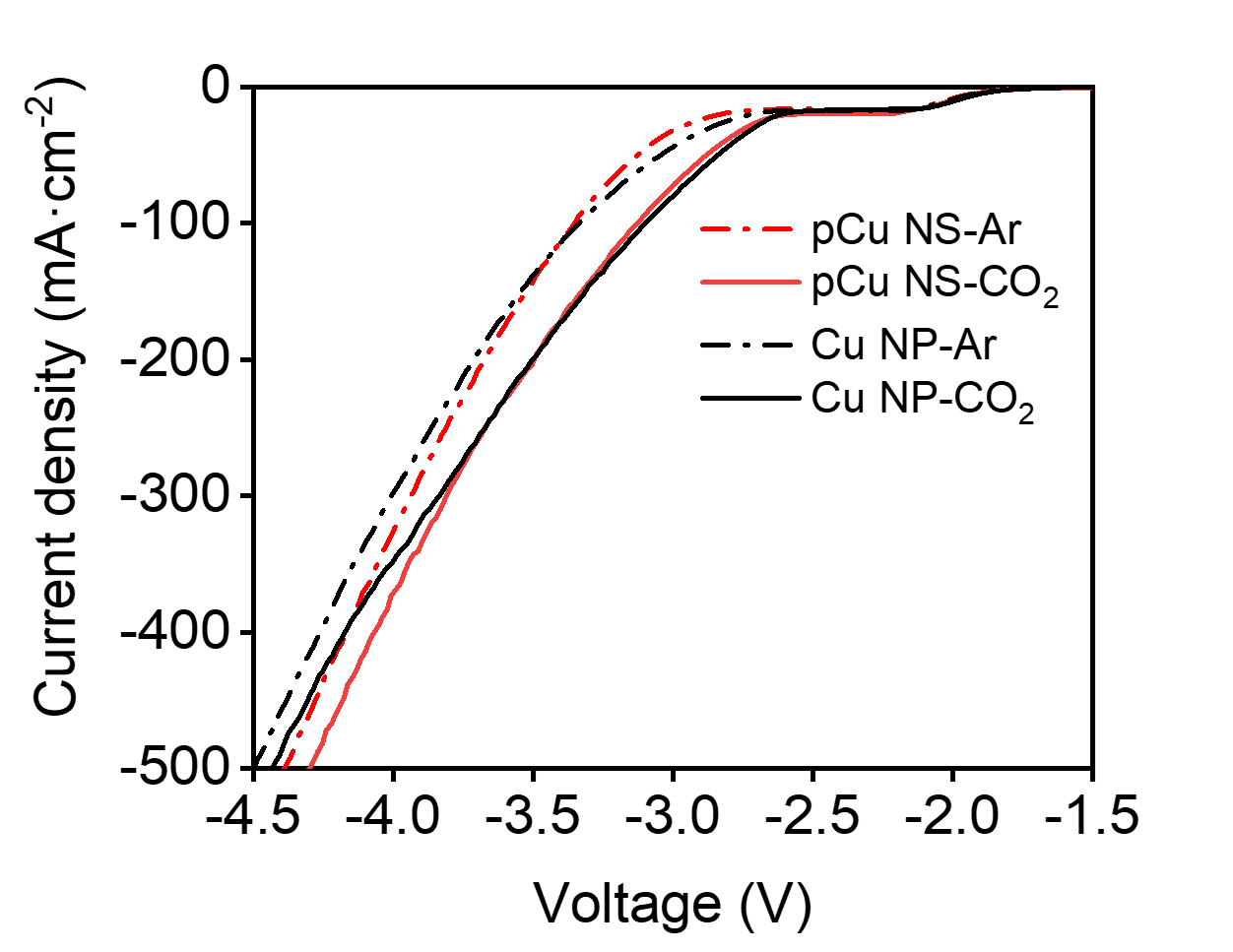
**

**Figure S17.** The LSV curves of pCu NS and Cu NP in the CO_2_ and Ar feed..

**
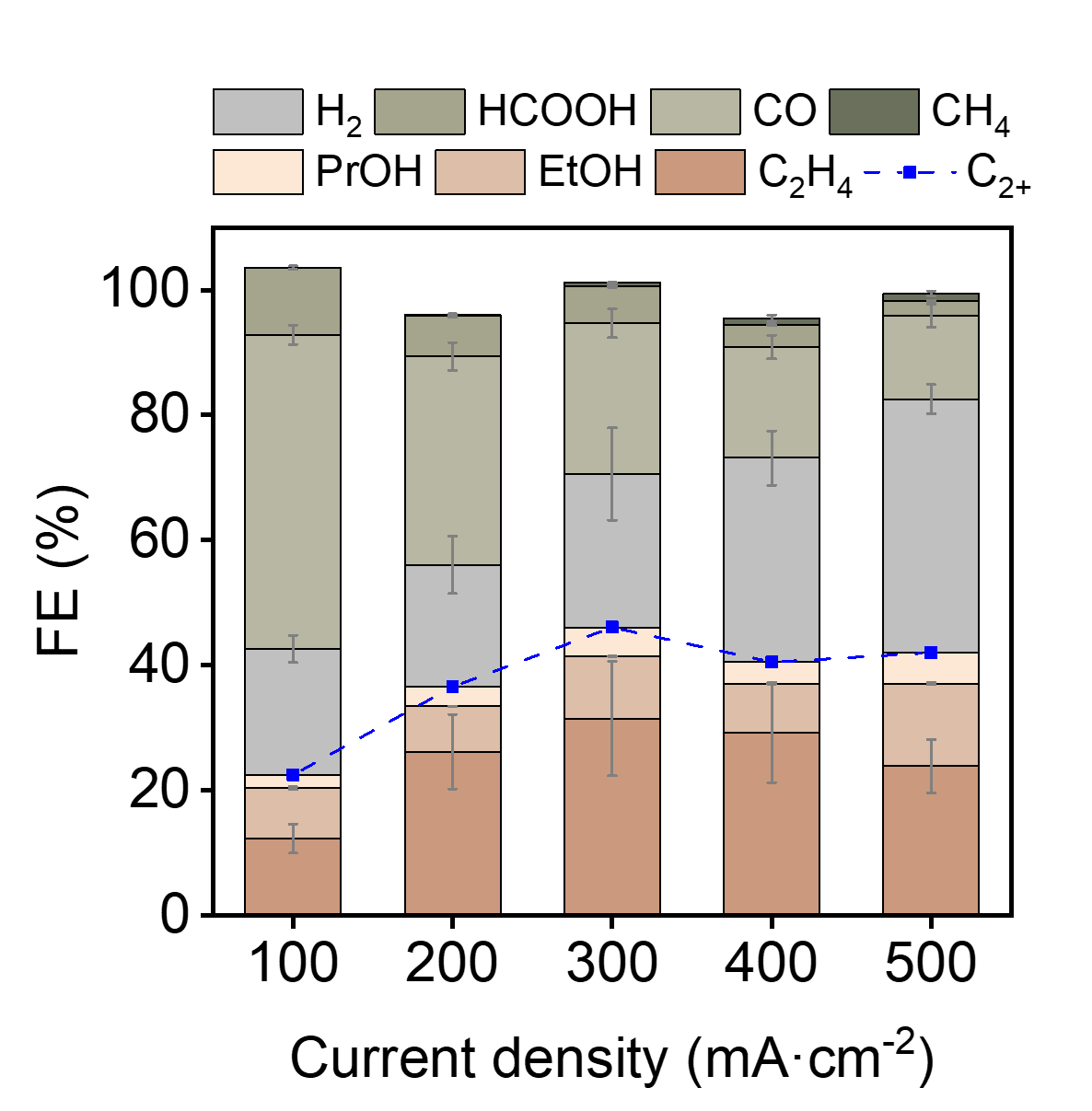
**

**Figure S18.** FEs under different current densities of Cu NP.

**
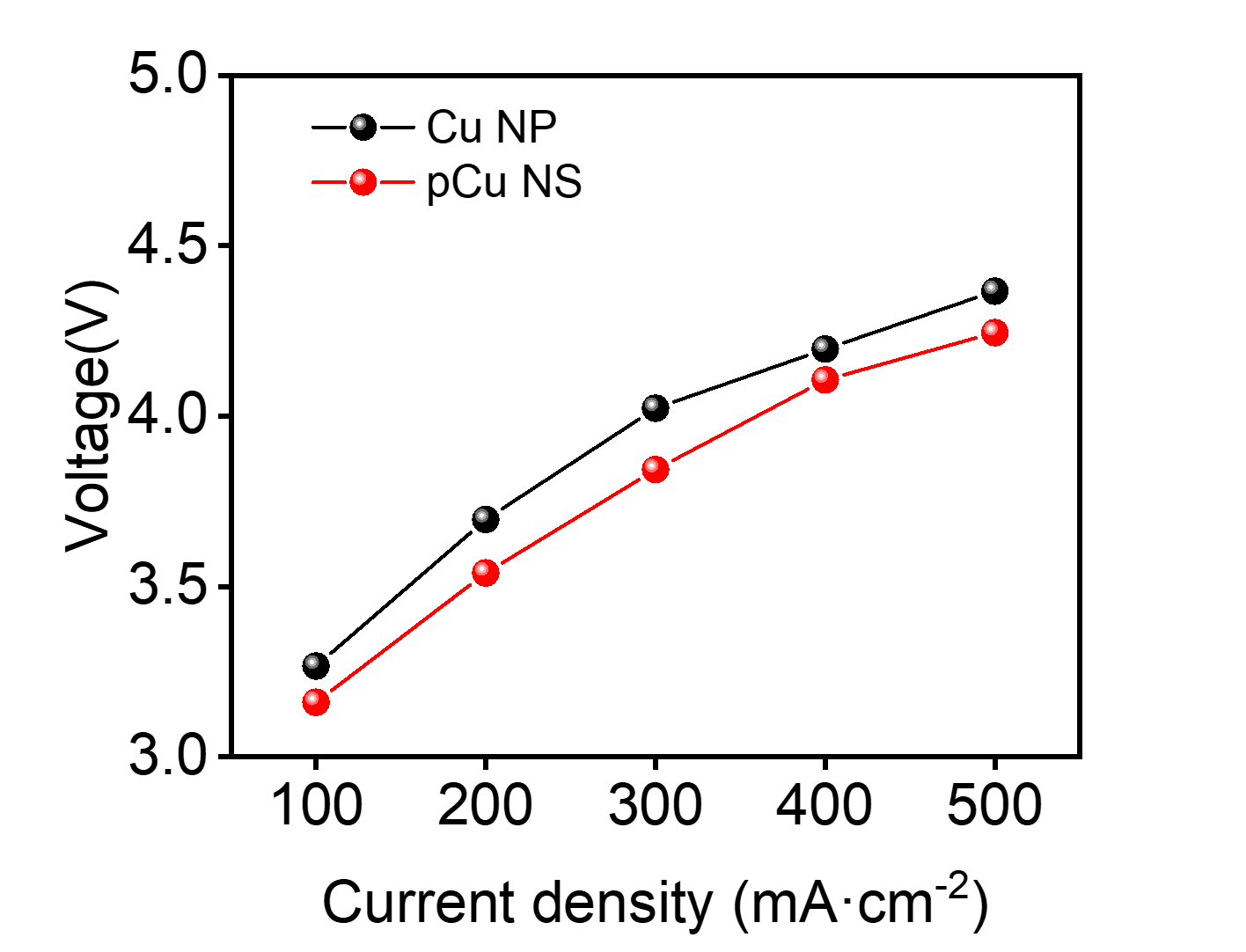
**

**Figure S19.** Cell voltage for pCu NS and Cu NP in different current densities.

**
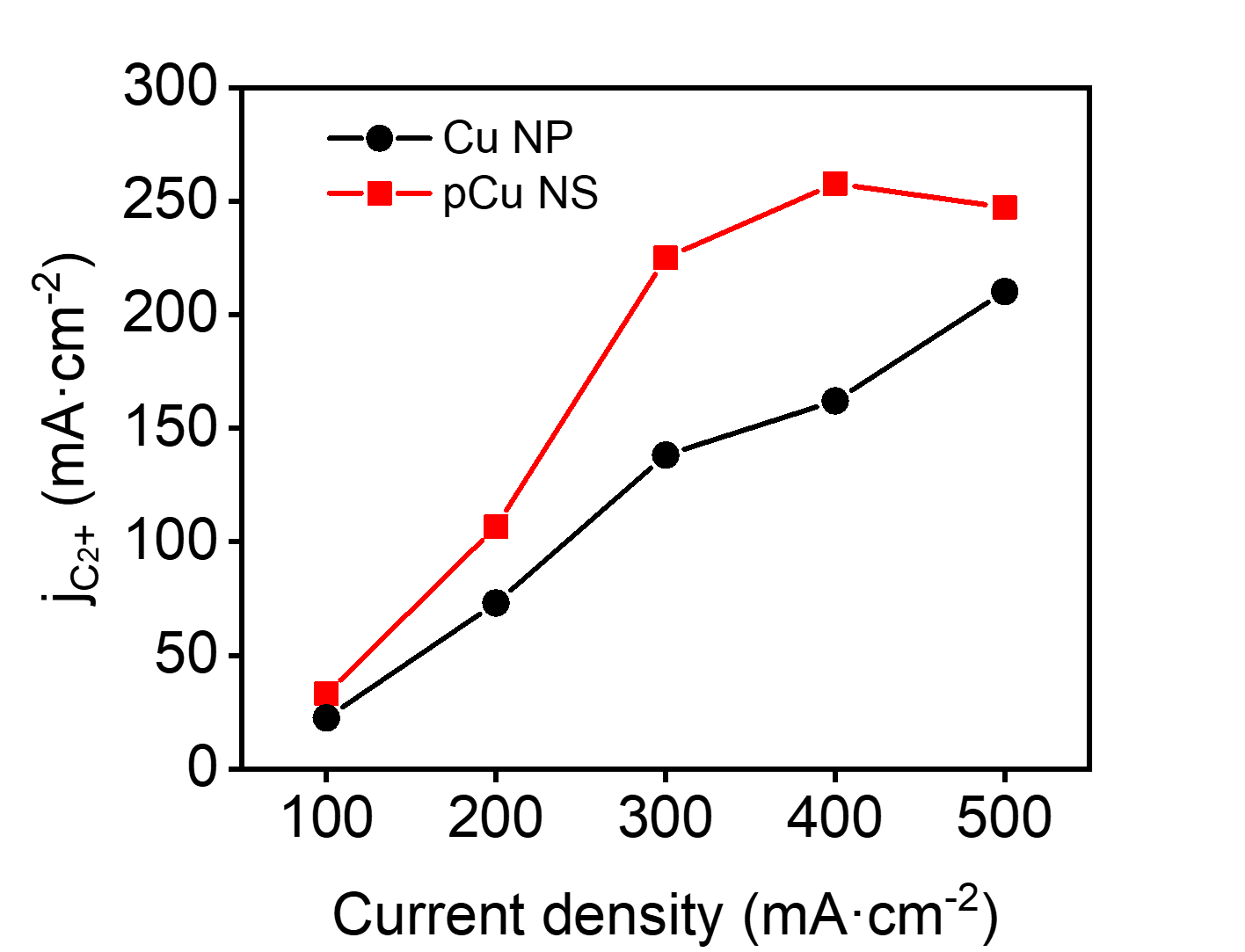
**

**Figure S20.** Partial current density of C_2+_ products of pCu NS and Cu NP.

**
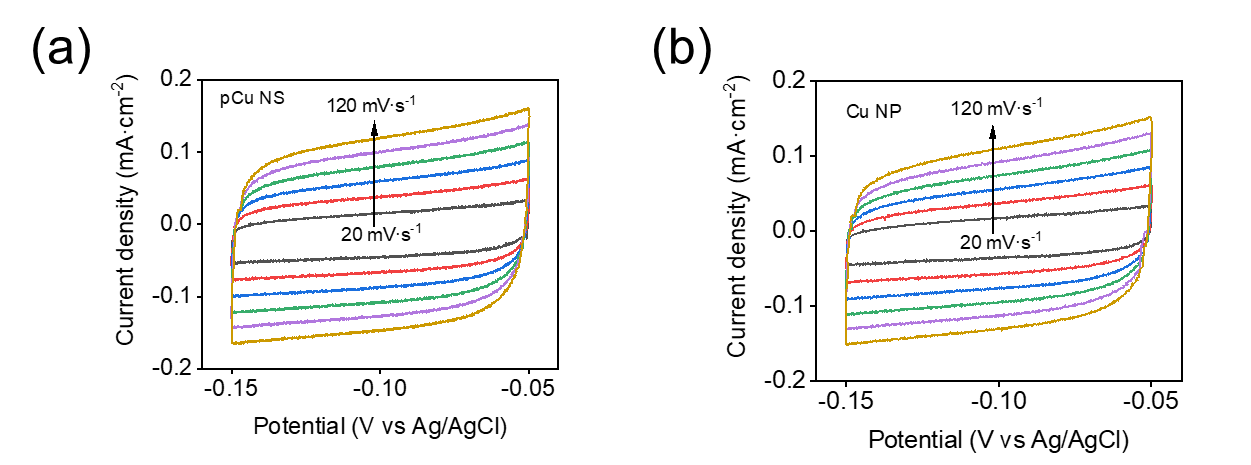
**

**Figure S21.** CV curves in a potential range of -0.15 to -0.05 V vs. Ag/AgCl for (a) pCu NS and (b) Cu NP.


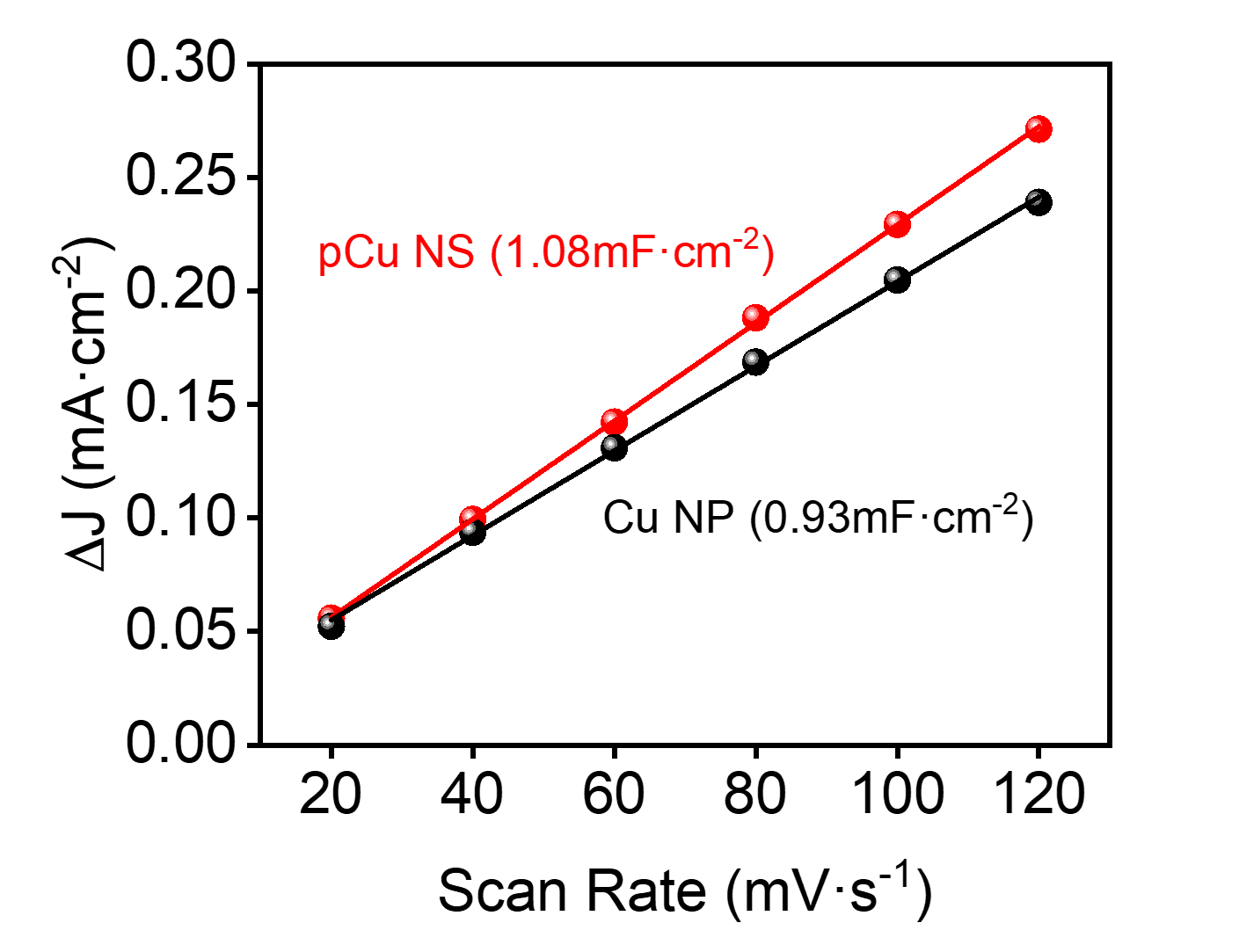


**Figure S22.** Cdl values for pCu NS and Cu NP.


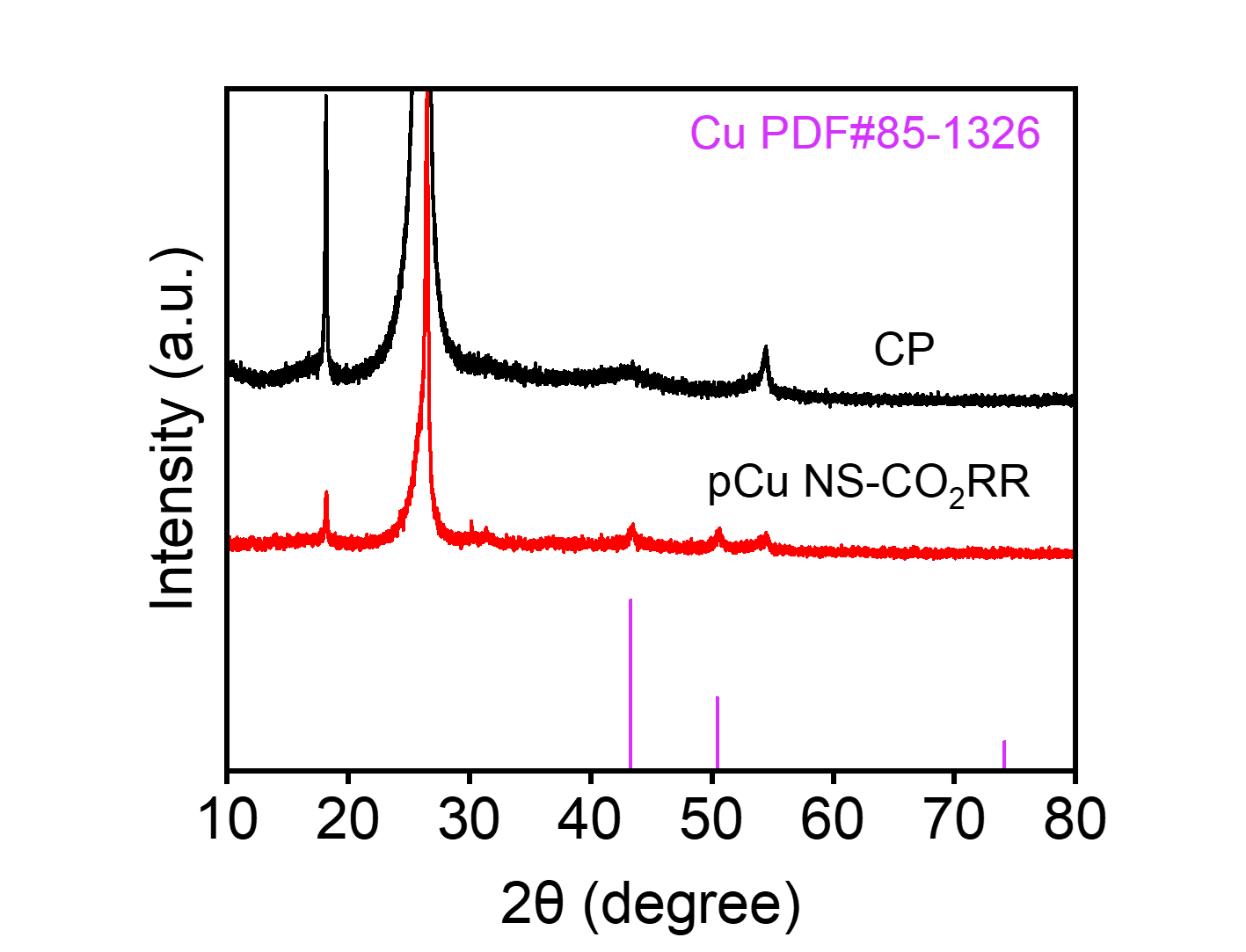


**Figure S23.** XRD images of pCu NS after CO_2_RR.


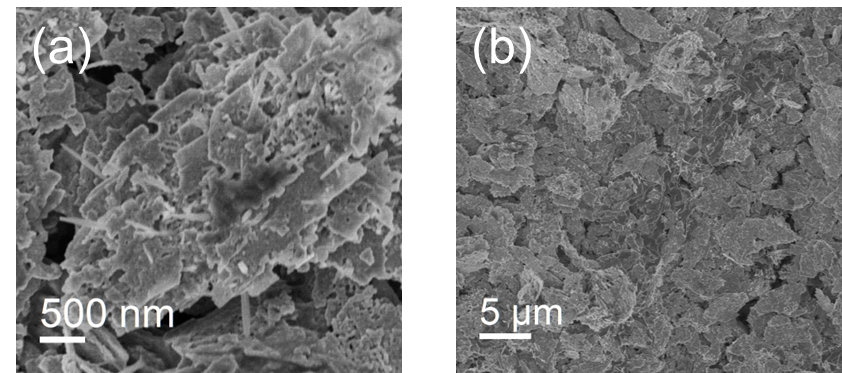


**Figure S24.** SEM images of (a-b) pCu NS after CO_2_RR.


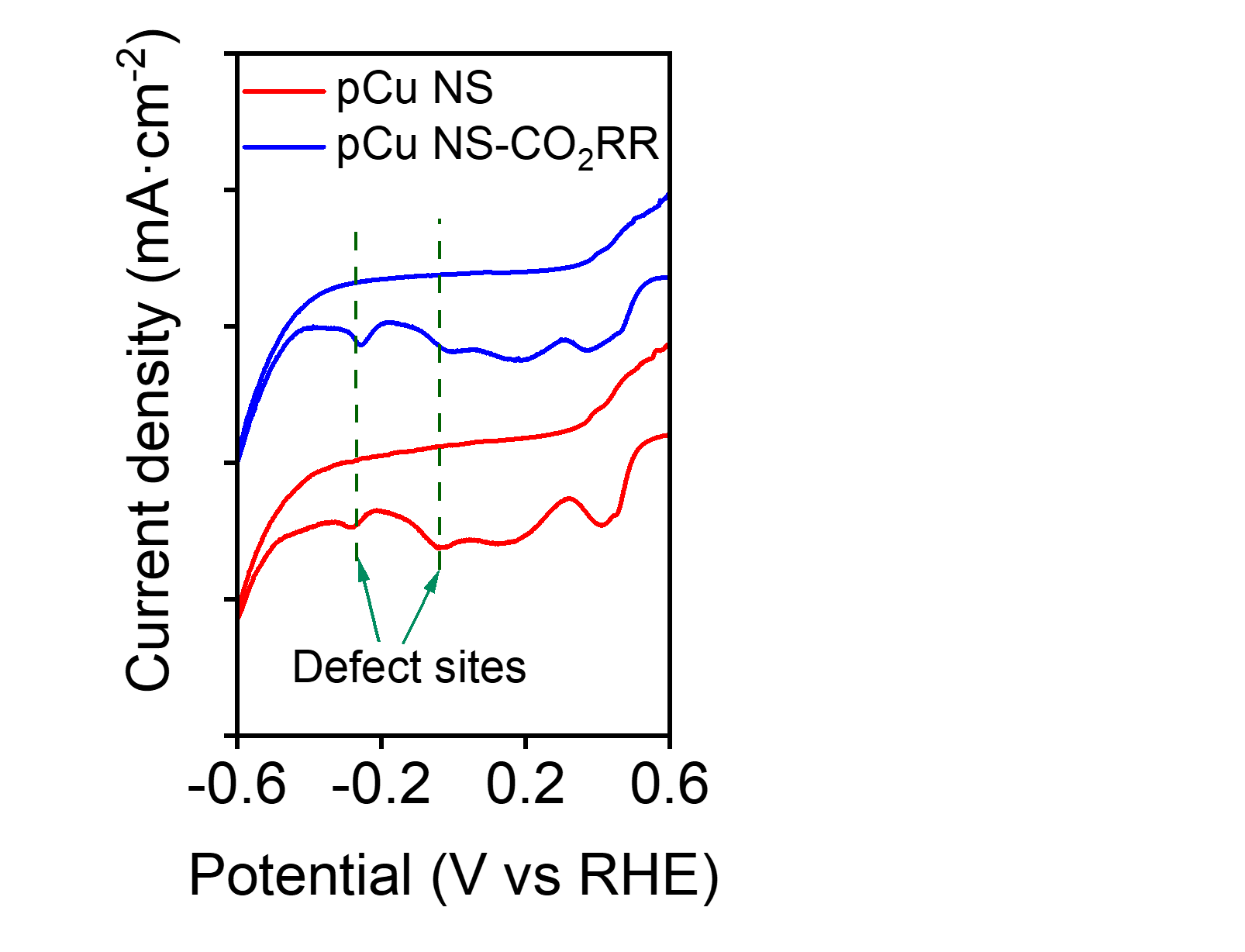


**Figure S25.** Cyclic voltammograms in Ar-saturated 0.1 M KHCO_3_ aqueous solution of as-prepared pCu NS and pCu NS after stability testing.


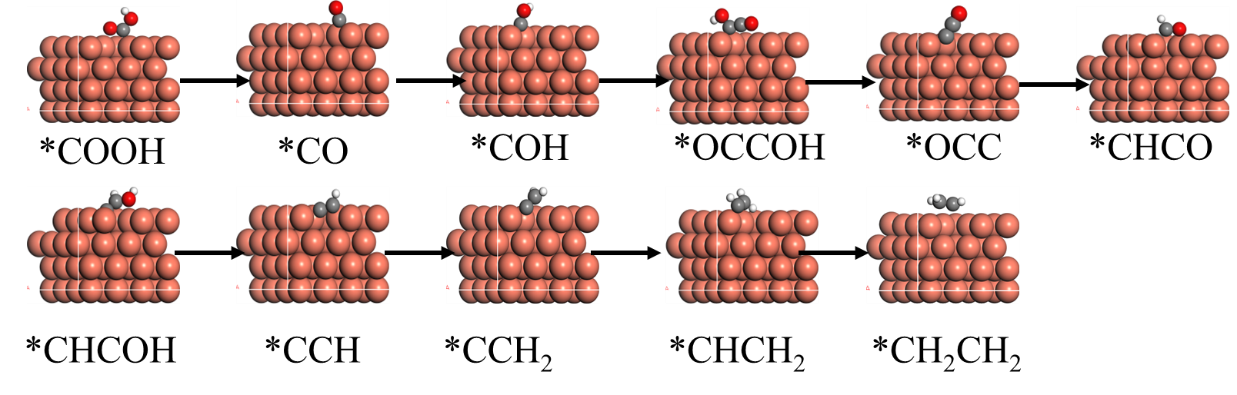


**Figure S26.** The reaction pathways of intermediates during ethylene (C_2_H_4_) formation on D-Cu (111).

**Table S1.** EXAFS fitting parameters at the Cu *K*-edge for various samples.

| Sample | Shell | CN^a^ | R(Å)^b^ | σ^2^(Å^2^)^c^ | ΔE_0_(eV)^d^ | K-range/Å^-1^ | R-range/Å | R factor |
| --- | --- | --- | --- | --- | --- | --- | --- | --- |
| Cu foil | Cu-Cu | 12* | 2.54±0.01 | 0.0085±0.0005 | 4.3±0.3 | 3.0-12.0 | 1.0-3.0 | 0.0037 |
| Cu2O | Cu-O | 2* | 1.84±0.01 | 0.0013±0.0008 | 6.6±0.6 | 3.0-12.0 | 1.0-3.0 | 0.0090 |
|  | Cu-Cu | 12* | 3.03±0.01 | 0.0193±0.0011 | 8.1±0.4 |  |  |  |
|  | Cu-O | 6* | 3.55±0.01 | 0.0130±0.0042 |  |  |  |  |
| pCu NS | Cu-Cu | 8.0±0.6 | 2.54±0.01 | 0.0080±0.0007 | 3.9±0.4 | 3.0-12.0 | 1.0-3.0 | 0.0082 |
| Cu NP | Cu-Cu | 8.8±0.6 | 2.54±0.01 | 0.0080±0.0006 | 4.3±0.3 | 3.0-12.0 | 1.0-3.0 | 0.0059 |

*^a^CN*, coordination number; *^b^R*, the distance to the neighboring atom; *^c^σ*^2^, Debye-Waller factor , the Mean Square Relative Displacement (MSRD); *^d^ΔE*_0_, inner potential correction; *R* factor indicates the goodness of the fit. *S*0^2^ was fixed to 0.875, accourding to the experimental EXAFS fit of Cu foil by fixing *CN* as the known crystallographic value. * This value was fixed during EXAFS fitting, based on the known structure of Cu. Error bounds that characterize the structural parameters obtained by EXAFS spectroscopy were estimated as CN ± 20%; R ± 1%; σ2 ± 20%; ΔE0 ± 20%. A reasonable range of EXAFS fitting parameters: 0.700 < *Ѕ*_0_^2^ < 1.000; *CN >* 0; *σ*^2^ > 0 Å^2^; |Δ*E*_0_| < 15 eV; *R* factor < 0.02.

**Table S2.** Comparison of catalyst with reported materials in acidic CO_2_RR.

| **Catalysts** | **Cell** | **FE_C2+ ()_** | **SPCE (%)** | **References** |
| --- | --- | --- | --- | --- |
| pCu NS | MEA | 75.01 | 74.38 | This work |
| CAL-modified Cu | Flow Cell | 43.9 | 77 | ^[5]^ |
| Pd–Cu | Flow Cell | 89 | 60 | ^[6]^ |
| Pd-doped Cu/Cu_2_O | Flow Cell | 64 | 73 | ^[7]^ |
| Cu/PTFE | MEA | 55 | 85 | ^[8]^ |
| Cu-GDL | Flow Cell | 87 | 42 | ^[9]^ |
| Cu hollow fiber | Flow Cell | 73.4 | 51.8 | ^[10]^ |
| Cu nanoneedle | Flow Cell | 90.69 | 25.49 | ^[11]^ |
| Porous Cu nanosheets | Flow Cell | 83.7 | 54.4 | ^[12]^ |

**Table S3.** Reaction path and Gibbs free energy barriers of CO_2_RR.

| Reaction Step | ∆G (eV) | | |
| --- | --- | --- | --- |
|  | D-Cu(100) | D-Cu(111) | Cu(100) |
| Slab + CO_2_(g) + H^+^ + e^-^ → *COOH | 0.33 | 0.44 | 0.56 |
| *COOH + H^+^ + e^-^ → *CO + H_2_O(g) | -0.38 | -0.58 | -0.76 |
| *CO + H^+^ + e^-^ → *COH | 0.64 | 1.53 | 0.76 |
| *CO + *COH→ *OCCOH | 1.12 | 0.82 | 1.15 |
| *OCCOH + H^+^ + e^-^ → *OCC + H_2_O(g) | -1.09 | -0.38 | -1.22 |
| *OCC + H^+^ + e^-^ → *OCCH | 0.04 | 0.15 | 0.52 |
| *OCCH + H^+^ + e^-^ → *CCHOH | 0.34 | 0.15 | -0.07 |
| *CCHOH + H^+^ + e^-^ → *CCH + H_2_O(g) | -0.34 | -0.72 | -0.23 |
| *CCH + H^+^ + e^-^ → *CCH_2_ | -0.38 | 0.06 | -0.48 |
| *CCH_2_+ H^+^ + e^-^ → *CHCH_2_ | -0.55 | -0.83 | 0.17 |
| *CHCH_2_+ H^+^ + e^-^ → *CH_2_CH_2_ | -0.28 | -0.54 | -0.75 |

**References**

[1] K. Momma, F. Izumi, VESTA 3 for three-dimensional visualization of crystal, volumetric and morphology data. *Applied Crystallography* **2011**, 44, 1272. https://doi.org/10.1107/S0021889811038970

[2] V. Wang, N. Xu, J.-C. Liu, G. Tang, W.-T. Geng, VASPKIT: A user-friendly interface facilitating high-throughput computing and analysis using VASP code. *Computer Physics Communications* **2021**, 267, 108033.https://doi.org/10.1016/j.cpc.2021.108033

[3] D. Sheppard, P. Xiao, W. Chemelewski, D. D. Johnson, G. Henkelman, A generalized solid-state nudged elastic band method. *The Journal of Chemical Physics* **2012**, 136, 074103. https://doi.org/10.1063/1.3684549

[4] R. A. Olsen, G. J. Kroes, G. Henkelman, A. Arnaldsson, H. Jónsson, Comparison of methods for finding saddle points without knowledge of the final states. *The Journal of Chemical Physics* **2004**, 121, 9776. https://doi.org/10.1063/1.1809574

[5] J. E. Huang, F. Li, A. Ozden, et al., CO_2_ electrolysis to multicarbon products in strong acid. *Science* **2021**, 372, 1074. https://doi.org/10.1126/science.abg6582

[6] Y. Xie, P. Ou, X. Wang, et al., High carbon utilization in CO_2_ reduction to multi-carbon products in acidic media. *Nature Catalysis* **2022**, 5, 564.

https://doi.org/10.1038/s41929-022-00788-1

[7] B. Wang, L. Song, C. Peng, X. Lv, G. Zheng, Pd-induced polarized Cu^0^-Cu^+^ sites for electrocatalytic CO_2_-to-C_2+_ conversion in acidic medium. *Journal of Colloid and Interface Science* **2024**, 671, 184. https://doi.org/10.1016/j.jcis.2024.05.156

[8] C. P. O’Brien, R. K. Miao, S. Liu, et al., Single Pass CO_2_ Conversion Exceeding 85% in the Electrosynthesis of Multicarbon Products via Local CO_2_ Regeneration. *ACS Energy Letters* **2021**, 6, 2952. https://doi.org/10.1021/acsenergylett.1c01122

[9] M. Sun, J. Cheng, M. Yamauchi, Gas diffusion enhanced electrode with ultrathin superhydrophobic macropore structure for acidic CO_2_ electroreduction. *Nature Communications* **2024**, 15, 491. https://doi.org/10.1038/s41467-024-44722-4

[10] C. Zhu, G. Wu, A. Chen, et al., Selective CO_2_ electroreduction to multicarbon products exceeding 2 A cm^−2^ in strong acids via a hollow-fiber Cu penetration electrode. *Energy & Environmental Science* **2024**, 17, 510. https://doi.org/10.1039/D3EE02867D

[11] X. Zi, Y. Zhou, L. Zhu, et al., Breaking K+ Concentration Limit on Cu Nanoneedles for Acidic Electrocatalytic CO_2_ Reduction to Multi-Carbon Products. *Angewandte Chemie International Edition* **2023**, 62, e202309351.

https://doi.org/https://doi.org/10.1002/anie.202309351

[12] Z. Ma, Z. Yang, W. Lai, et al., CO_2_ electroreduction to multicarbon products in strongly acidic electrolyte via synergistically modulating the local microenvironment. *Nature Communications* **2022**, 13, 7596. https://doi.org/10.1038/s41467-022-35415-x
